# Clinical Effectiveness of Ritonavir-Boosted Nirmatrelvir—A Literature Review

**DOI:** 10.3390/arm92010009

**Published:** 2024-01-18

**Authors:** Sydney Paltra, Tim O. F. Conrad

**Affiliations:** 1 FG Verkehrssystemplanung und Verkehrstelematik, Technische Universität Berlin, 10623 Berlin, Germany; 2 Zuse Institute Berlin, Takustraße 7, 14195 Berlin, Germany; conrad@zib.de

**Keywords:** COVID-19, SARS-CoV-2, paxlovid, ritonavir, nirmatrelvir, nirmatrelvir/ritonavir

## Abstract

**Highlights:**

**What are the main findings?**

- Nirmatrelvir/Ritonavir, an oral treatment for COVID-19, effectively reduces the risk of progressing to a more severe disease state for both the Delta and subsequent Omicron variants.
- Unvaccinated COVID-19 patients benefit more from a Nirmatrelvir/Ritonavir prescription than vaccinated ones.

**What is the implication of the main finding?**

- Although effective at reducing the risk of progressing to a more severe disease state, Nirmatrelvir/Ritonavir cannot replace vaccinations.
- Future subanalyses should focus on pinpointing the role of age, strain, or comorbidities in effectiveness differences.

**Abstract:**

Nirmatrelvir/Ritonavir is an oral treatment for mild to moderate COVID-19 cases with a high risk for a severe course of the disease. For this paper, a comprehensive literature review was performed, leading to a summary of currently available data on Nirmatrelvir/Ritonavir’s ability to reduce the risk of progressing to a severe disease state. Herein, the focus lies on publications that include comparisons between patients receiving Nirmatrelvir/Ritonavir and a control group. The findings can be summarized as follows: Data from the time when the Delta-variant was dominant show that Nirmatrelvir/Ritonavir reduced the risk of hospitalization or death by 88.9% for unvaccinated, non-hospitalized high-risk individuals. Data from the time when the Omicron variant was dominant found decreased relative risk reductions for various vaccination statuses: between 26% and 65% for hospitalization. The presented papers that differentiate between unvaccinated and vaccinated individuals agree that unvaccinated patients benefit more from treatment with Nirmatrelvir/Ritonavir. However, when it comes to the dependency of potential on age and comorbidities, further studies are necessary. From the available data, one can conclude that Nirmatrelvir/Ritonavir cannot substitute vaccinations; however, its low manufacturing cost and easy administration make it a valuable tool in fighting COVID-19, especially for countries with low vaccination rates.

## 1. Introduction

Over the last three years, health officials have faced challenges of unknown dimensions from the COVID-19 pandemic. By 2023, the pandemic was responsible for more than 769 M cases and more than 6.9 M deaths [1]. Beyond enormously straining healthcare systems, societies also faced psychological and socio-economical challenges [2,3,4], often affecting minorities the most [5]. Hence, treating the infected and curbing spread remains paramount.

In fighting the pandemic, much hope rested on effective vaccines. Currently, the EU has authorized eight [6], the UK has authorized eight [7], and the USA has authorized three vaccines [8]. A recent study [9] estimated vaccines to have saved 14–20 million lives during the first year of the vaccine roll-out. However, the distribution of vaccines has been uneven globally, and their effectiveness can wane over time. Moreover, not everyone is medically able or willing to receive a vaccination. Consequently, in addition to vaccine efforts, significant scientific resources have been devoted to developing antiviral medications. The currently available treatments include:

- Remdesivir (Veklury) [10] is the first available treatment for COVID-19. Countries that authorized the administration of Remdesivir include Singapore, the EU, the USA, the UK, and Australia. Treatment must be administered within seven days of symptom appearance, intravenously, and the treatment duration varies by the patient’s state.
- Molnupiravir (Lagevrio) [11] is taken orally at home. Molnupiravir was authorized, for example, in the UK in November 2021 and by the FDA (USA) in December 2021. It must be administered within five days of symptom appearance and taken every 12 h over five days.
- Sotrovimab (Xevudy) [12,13] is a neutralizing monoclonal antibody (nMAb). Sotrovimab is available for treatment in the EU, the UK, Australia, and Singapore, among others. Administration must start within 5 days of symptom appearance and be administered through an intravenous infusion.

In addition to these treatments, Nirmatrelvir/Ritonavir has emerged as a noteworthy option for the treatment and post-exposure prophylaxis of COVID-19. This antiviral combination consists of Nirmatrelvir tablets, which target the SARS-CoV-2 M^pro^ protease enzyme to hinder viral replication, and Ritonavir tablets, a protease inhibitor that enhances the pharmacokinetics of Nirmatrelvir by slowing its breakdown in the liver. This synergy allows Nirmatrelvir to achieve higher systemic concentrations [14]. Nirmatrelvir/Ritonavir’s recommended dosage consists of two 150 mg Nirmatrelvir tablets and one 100 mg Ritonavir tablet, taken orally together, twice daily for five consecutive days [15,16]. It is important to complete the five-day treatment, but usage beyond this duration is not authorized. Potential side effects of Nirmatrelvir/Ritonavir include an impaired sense of taste, diarrhea, high blood pressure, and muscle aches, with caution for possible drug-drug interactions when taken with other medication [15]. However, overall Nirmatrelvir/Ritonavir represents a significant advancement in the therapeutic arsenal against COVID-19, given its ease of administration and effectiveness, especially in scenarios where vaccination is not feasible or insufficient.

Furthermore, the significance of Nirmatrelvir/Ritonavir in reducing healthcare burdens cannot be overstated. Despite the ongoing challenges posed by varying infection rates and the emergence of new strains, the presence of a convenient and efficient oral medication plays a crucial role in effectively managing the pandemic. The accessibility and cost-effectiveness of this tool are especially important in areas with low healthcare resources, making it a valuable asset in global endeavors to combat COVID-19.

In this paper, a review of the available literature is conducted that focuses on the effectiveness of Nirmatrelvir/Ritonavir in preventing the severe progression of COVID-19, with a particular emphasis on comparing its impact on patients receiving the treatment to a control group. The discussion synthesizes findings across various studies, considering factors such as vaccination status, study design variation, and the role of different COVID-19 variants, to provide a comprehensive understanding of the role of Nirmatrelvir/Ritonavir in the ongoing fight against the pandemic.

## 2. Methods

In conducting this systematic review, our objective was to rigorously evaluate the available scientific literature regarding the efficacy of Nirmatrelvir/Ritonavir in treating COVID-19. To achieve a high level of rigor and transparency, we adopted the Preferred Reporting Items for Systematic Reviews and Meta-Analyses (PRISMA) guidelines.

Search Strategy and Selection Criteria: To ensure a comprehensive and systematic review, we followed the PRISMA guidelines. We conducted a detailed search on PubMed and MedRxiv for publications containing at least one of the following terms in their title or abstract: “Paxlovid”, “Nirmatrelvir”, or “Ritonavir”. The search was limited to publications dated up to 31 July 2023.

Screening Process: Initially, our search yielded a total of 362 publications (see Supplementary Materials). These were subjected to a two-stage screening process. In the first stage, the abstracts were reviewed for relevance to our specific research topic: assessing the effectiveness of Nirmatrelvir/Ritonavir in reducing the risk of severe COVID-19 progression.

Inclusion and Exclusion Criteria: For inclusion, studies needed to directly compare the outcomes of patients treated with Nirmatrelvir/Ritonavir to a control group that did not receive the antiviral treatment. We excluded studies that lacked an untreated control cohort, such as study [17], to maintain the integrity of our comparative analysis.

Study Selection: Following this rigorous screening process, 17 studies were deemed relevant and met our inclusion criteria. These studies form the basis of our review, providing a focused and comprehensive examination of Nirmatrelvir/Ritonavir’s effectiveness.

## 3. Review

In the dynamic field of COVID-19 research, an accurate understanding needs a combination of extensive scope and profound analysis. To offer a more structured and practical analysis, a result-driven synthesis is given first, emphasizing specific outcomes linked to the utilization of Nirmatrelvir/Ritonavir. This facilitates a straightforward evaluation of the drug’s effects across multiple studies, with a focus on important outcomes such as hospitalization or mortality. Based on this synthesized perspective, the studies included in this review are presented in more detail, providing an extensive examination of the methodologies and results within their respective contexts.

Hospitalization: Numerous studies have demonstrated the effectiveness of Nirmatrelvir/Ritonavir in decreasing the rates of hospitalization. For example, several studies [18,19,20,21] have documented substantial reductions in relative risk, ranging from 26% to 88.9% (for hospitalization or death). Several studies [22,23,24,25] consistently reported a decrease in the likelihood of hospitalization among individuals treated with the drug. The odds and hazard ratios consistently indicated a favorable outcome for those who received the treatment. Furthermore, the study [26] underscored a decreased likelihood of overnight hospital admissions among the recipients.

Death: The efficacy of Nirmatrelvir/Ritonavir in reducing mortality has been consistently demonstrated in multiple studies. In [18,19,21], a relative risk reduction of 100% was observed. Other studies [20,23,24,27,28,29] consistently demonstrated a decrease in mortality risks, thereby highlighting the potential of the drug in terms of life-saving capabilities.

ICU Admissions: The effect of Nirmatrelvir/Ritonavir on admissions to the Intensive Care Unit (ICU) was also significant. The findings in [30] indicate that patients who received treatment with the drug exhibited a decrease in the number of admissions to the ICU. Cai et al. [28] provide additional support for this observation.

Other: In addition to the primary outcomes, several studies have investigated additional advantages associated with the administration of Nirmatrelvir/Ritonavir. There is evidence that the relative risk of emergency room visits decreases significantly [19,21]. Cegolon et al. [24] demonstrated the drug’s influence on viral clearance, as indicated by a higher rate of “negativization”. Moreover, Wong et al. [29] highlighted the fact that individuals who utilized antiviral treatments experienced a more rapid reduction in viral load. Nevertheless, it is important to acknowledge that Liu et al. [31] observed no statistically significant differences in specific outcomes when comparing a treatment with a control group.

Given the preceding presentation of the summary focused on outcomes, an elaborate account of each study is provided below, enabling readers to gain a comprehensive understanding of the specific research conducted in the respective studies.

The first study considered in this review is a phase 2–3, double-blind, randomized, placebo-controlled trial [18] with patients from 21 countries (see Table 1 for the main results of this section and Table S1 for a summary of the characteristics of the considered studies). Only adult unvaccinated patients with a symptom onset of no more than five days were eligible. This resulted in 2246 patients, who were randomly split into two groups: 1120 received Nirmatrelvir/Ritonavir and 1126 received a placebo. The final analysis at the end of the 28-day follow-up period included only patients who had commenced treatment within three days of symptom onset. Five of the 697 patients in the treated group and 44 of the 682 in the placebo group were hospitalized for COVID-19 or died from any cause, which corresponds to an 88.9% relative risk reduction. During the trial study period, Delta prevailed as the dominant variant, whereas all subsequent studies focused on the period of Omicron’s dominance. Furthermore, Hammond et al. reported that the adverse events that were considered to be related to the drug/placebo occurred more often among participants receiving Nirmatrelvir/Ritonavir (7.8%) in comparison with participants receiving the placebo (3.8%). Nine participants in the Nirmatrelvir/Ritonavir and seven participants in the control group discontinued treatment due to adverse events. The side effects most commonly reported by Nirmatrelvir/Ritonavir recipients were dysgeusia, diarrhea, fibrin D-dimer increase, alanine aminotransferase increase, nausea, and vomiting.

In [21], the authors searched the TriNetX research network for data. Vaccinated adult patients who developed COVID-19 at least one month after their vaccination and between 1 December 2021 and 18 April 2022 were included. They identified 111,588 non-hospitalized patients during the study period, of whom 1131 had received Nirmatrelvir/Ritonavir. Using propensity score matching, they defined the treated and the control cohort, consisting of 1130 patients each. At a follow-up 30 days after diagnosis, the studied primary outcomes were all-cause emergency room (ER) visits, hospitalization, or death. The relative risk reduction for all composite outcomes was 45%; the authors furthermore identified a relative risk reduction of 41% for ER visits, 60% for hospitalization, and 100% for death.

The retrospective cohort study by [19] compared two propensity-matched cohorts of 2547 patients each, assembled from the TriNetX database. Eligible patients had to be 50 or younger, vaccinated for at least one month, and diagnosed with COVID-19 between 1 December 2021 and 30 July 2022. At the 30-day follow-up, the authors found a reduced risk for all emergency room visits (odds ratio (OR) 0.818, RR 0.8333), hospitalization (OR 0.345, relative risk (RR) 0.3529), and 30-day mortality (OR not provided, RR 0), as well as a reduced risk for the corresponding composite outcome (OR 0.683, RR 0.7). Additionally, subgroup analyses reported significantly lower ORs for the primary composite outcomes for patients suffering from cancer (OR 0.692), cardiovascular disease (OR 0.629), or cancer and cardiovascular disease (OR 0.432).

Hansen et al. [20] performed a multi-institute retrospective cohort study on the National COVID Cohort Collaborative (N3C) database in the USA. A total of 1,029,910 patients were deemed eligible for prescription of Nirmatrelvir/Ritonavir between December 2021 and February 2023, and 98,060 were treated with the drug within 5 days of diagnosis. Considering a 28-day follow-up period, the study found a reduced hospitalization risk (RR 0.742) and a reduced mortality risk (RR 0.269).

Bhatia et al. [22] analyzed electronic health record data from the N3C repository. A total of 410,642 adult patients with a positive test between 22 December 2021 and 31 December 2022 were considered. Of these, Nirmatrelvir/Ritonavir was administered to 104,510 patients, with 628 requiring hospitalization. In the control cohort, 5549 patients were hospitalized, leading to an adjusted odds ratio (aOR) of 0.33.

The authors of [27] use data from the Israeli Ministry of Health COVID-19 database. The study included adults with a first-ever positive COVID-19 test between 1 January 2022 and 28 February 2022, who had at least one comorbidity or condition associated with a high risk for severe COVID-19. The follow-up ended either (a) 28 days after diagnosis, (b) with the occurrence of severe COVID-19 or death, or (c) on 10 March 2022, whichever came first. Included were 180,351 patients. Of these, 4737 received Nirmatrelvir/Ritonavir, and 135,482 were vaccinated. The considered outcome was the composite of severe COVID-19 or mortality. The authors found that Nirmatrelvir/Ritonavir was independently associated with a significantly decreased risk for the composite of severe COVID-19 or mortality (hazard ratio (HR) 0.54).

In [25], data from a large US healthcare system was accessed. Individuals were included if they (a) were over 50 years old, (b) received a COVID-19 diagnosis between 1 January 2022 and 17 July 2022, and (c) did not test PCR-positive in the 90 days before their diagnosis. Exclusions encompassed administration of alternative COVID-19 treatments or medication deemed incompatible with Nirmatrelvir/Ritonavir and diagnosis upon hospitalization/death. This resulted in 44,551 eligible outpatients, of whom 12,541 were prescribed Nirmatrelvir/Ritonavir. The considered primary outcome was hospitalization within 14 days or death within 28 days of infection. In the treated group, 69 were hospitalized or died, while in the control group, 310 were hospitalized or died, leading to an adjusted risk ratio (aRR) of 0.56. The treated group had a lower risk for hospitalization (aRR 0.60) and death (aRR 0.29).

The retrospective matched cohort study [30] included all adult patients who were treated with Nirmatrelvir/Ritonavir in two Saudi-Arabian hospitals in 2022. Overall, 92 patients were considered, of whom 28 received Nirmatrelvir/Ritonavir. The study found that the intake of Nirmatrelvir/Ritonavir was associated with lower ICU admissions (0% vs. 18.75%) and lower deaths (3.57% vs. 26.56%), although the authors pointed out that in the treated group, there were fewer immunocompromised patients.

A second study, using data from 109,254 patients from the Israeli Clalit Health Services database [23], considered the period from 9 January 2022 to 10 March 2022. Eligible patients had to have tested positive by 31 March 2022, be at least 40 years old, have a confirmed COVID-19 infection, be considered high-risk for severe disease, and be deemed eligible for Nirmatrelvir/Ritonavir therapy. Excluded were patients residing in a long-term care facility, patients who were hospitalized during the study period but before a positive SARS-CoV-2 test result, and patients treated with Molnupiravir. A patient’s follow-up ended either (a) 35 days after diagnosis, (b) at the end of the study period, or (c) if censored due to death, whichever came first. The authors reported results for two separate age groups: 40–64 and 65+. Of the 42,821 eligible patients aged 65 years and older, 2483 were treated with Nirmatrelvir/Ritonavir. In this age group, the treated cohort showed a lower risk for hospitalization (aHR 0.27) and death (aHR 0.19). For people aged 40–64, the authors found an aHR of 0.74 (hospitalization) and 1.32 (death).

In the retrospective controlled clinical study [24], 386 high-risk COVID-19 outpatients in Italy, diagnosed between 1 February 2022 and 31 May 2022, were considered. Of the 386 considered patients, 116 were treated with Molnupiravir, 102 were treated with Nirmatrelvir/Ritonavir, 57 were treated with Sotrovimab, and 111 received standard care. Within the 30-day follow-up period, the treated cohorts had a lower risk of hospital admission (N/R: aOR 0.16, SOT: aOR 0.22, MOL omitted, as no patient receiving Molnupiravor was hospitalized) and a lower mortality risk (two people from the control group, none from the treated groups died as a result of COVID-19). Furthermore, the “negativization rate” was higher for the recipients of Nirmatrelvir/Ritonavir and Molnupiravor (N/R: aOR 1.68, MOL: 1.45) but lower for recipients of Sotrovimab (aOR 0.86).

Within a study period between 6 February 2022 and 2 April 2022, Park et al. [33] considered 2241 patients and workers at five long-term care facilities in South Korea. Among the confirmed cases in these facilities, 44.7% of the patients and 0.2% of the workers received oral treatment. Nirmatrelvir/Ritonavir was the preferred treatment, making up 86.6% of all oral treatments. The authors found a lower risk for severe illness or death among the treated (aRR 0.49) and a lower risk for death itself (aRR 0.62).

A retrospective cohort study on hospitalized patients in Hong Kong was published by Wong et al. [29]. The authors considered patients who were diagnosed between 26 February 2022 and 26 April 2022. Taking data from 40,776 hospitalized but non-oxygen-dependent patients into account, they matched oral antiviral users and controls using propensity-score matching in a 1:1 ratio. In consequence, 1856 Molnupiravir (Mol) users, 890 Nirmatrelvir/Ritonavir (N/R) users, and 2746 control patients over a mean follow-up of 41.3 days were included. The authors found that antiviral users had a significantly lower risk of all-cause mortality (Mol HR = 0.48, N/R HR = 0.24), the composite outcome of disease progression (Mol HR = 0.69, N/R HR = 0.57), and the need for oxygen therapy (Mol HR = 0.69, N/R HR = 0.73). Additionally, the time to achieve a lower viral burden was significantly shorter among antiviral users.

Complementarily, Cai et al. [28] focused on hospitalized COVID-19 patients with chronic kidney disease. The retrospective cohort study included hospitalized adults between 7 April 2022 and 21 June 2022 in Renji Hospital in Shanghai. Of the 1279 included patients, 469 received Nirmatrelvir/Ritonavir. Their primary outcome was all-cause death, for which the treated cohort displayed a lower risk (OR 0.4). Secondary outcomes included ICU admission and invasive ventilation. Again, the treated cohort displayed a lower risk (OR 0.49 and OR 0.93).

In their work, Liu et al. [31] considered hospitalized COVID-19 patients suffering from severe comorbidities (SCs). The study was an open-label, multicenter, random controlled trial on hospitalized adults with SCs who were eligible for Nirmatrelvir/Ritonavir treatment and who were assigned to a control cohort in a 1:1 ratio. At five sites in Shanghai, between 10 April 2022 and 19 May 2022, 264 patients were considered. The considered outcomes were 28-day mortality, hospital mortality, and non-invasive mechanical ventilation, for which no significant difference could be determined between the treatment and control groups. Furthermore, Liu et al. reported on the number of adverse events during the treatment period: For both groups, the number of adverse events was similar (17 (12.9%) in the Nirmatrelvir/Ritonavir group vs. 13 (9.8%) in the standard treatment group), as was the number of serious adverse events (six in the N/R group vs. five in the standard treatment group). However, four Nirmatrelvir/Ritonavir recipients had to discontinue the treatment because of adverse events vs. zero patients in the standard treatment group.

Shah et al. [26] published an analysis of data from a large electronic health record data set (COSMOS). In this data set, 699,848 adults were eligible for Nirmatrelvir/Ritonavir prescription from April to August 2022, of which 28.4% received a prescription within 5 days of diagnosis. Recipients had an overall lower risk of overnight hospital stay (aHR 0.49). A subgroup analysis strongly suggested that recipients who had also received at least two or three doses of an mRNA vaccine also had a lower hospitalization risk (aHR of 0.5 and aHR of 0.3), as did the treated groups of 18–49/50–64/65+-year-olds (aHR: 0.59, 0.4 and 0.53, respectively).

In [32], patients diagnosed between 1 July 2022 and 30 November 2022 in South Korea were considered. The nationwide retrospective study included 1,936,925 COVID-19 patients who were at least 12 years old, who contracted COVID-19 during the study period, and who were eligible for a Nirmatrelvir/Ritonavir prescription. Of these, 420,996 received the drug. The monitoring ended 28 days after the diagnosis, and the study indicated that drug recipients had a lower risk of severe/critical illness or death (OR 0.568), where the unvaccinated profited more from the drug intake than the vaccinated (OR of 0.46 vs. 0.782). Furthermore, their subgroup analyses of people aged 60+/70+/80+ revealed a lower risk of severe/critical illness or death (OR 0.540/0.537/0.551, respectively).

Shao et al. [34] considered 1082 severely and critically ill (hospitalized) patients in Shanghai from 8 December 2022 to 9 February 2023. They found a lower risk for 60-day mortality in the group of Azvudine recipients (aHR 0.44) and the group of Nirmatrelvir/Ritonavir recipients (aHR 0.71) compared with the group not receiving an antiviral but potentially an alternative form of therapy.

## 4. Discussion

In this review, the presented studies show varying but significant effectiveness for Nirmatrelvir/Ritonavir. This may be traced back to diverse study periods (initial clinical study during Delta and subsequent studies during Omicron dominance) and different study populations (clinical study included unvaccinated individuals only, while subsequent studies included vaccinated and unvaccinated individuals). Unvaccinated patients benefit more from a Nirmatrelvir/Ritonavir prescription, but further subanalyses are necessary to pinpoint the role of age, strain, or comorbidities in effectiveness differences. Furthermore, study design variations limit comparisons. Yet, Nirmatrelvir/Ritonavir has proven to remain effective for current variants by limiting the progression to a more severe disease state and having a positive effect regarding outcomes such as hospitalization or mortality. However, it shall be noted that this review solely focuses on Nirmatrelvir/Ritonavir’s ability to prevent disease progression. For prescription decisions, (a) Long COVID risk [35], (b) drug–drug interactions [36,37,38], (c) rebound effects [39,40,41], and (d) the potential danger of adverse events [42] should also be considered. Finally, we must point out the rising cost of Nirmatrelvir/Ritonavir administration and the uneven allocation of the drug worldwide [43,44], which were not discussed in this review, but which limit the drug’s potential in fighting this global healthcare crisis.

In light of the detailed results from the 17 studies analyzed, it is evident that Nirmatrelvir/Ritonavir offers substantial benefits across various demographics and clinical scenarios, particularly in reducing hospitalizations and mortality rates. These findings reinforce the potential of Nirmatrelvir/Ritonavir as a critical intervention in COVID-19 management, especially when vaccine access is limited or in cases where vaccination alone may not be sufficient. However, it is important to note that the effectiveness of the treatment varies depending on the viral variant, patient demographics, and underlying health conditions, underscoring the need for personalized medical approaches in managing COVID-19.

Altogether, it may be concluded that Nirmatrelvir/Ritonavir is a valuable tool, especially in countries with a low vaccination rate, but it cannot replace vaccines and is not a panacea in the fight against the COVID-19 pandemic.

## Figures and Tables

**Table 1:** Efficacy results from the 17 studies presented in this review. Here, an event is the occurrence of the studied outcome. [24,28,29] considered hospitalized patients, whereas all the remaining studies considered non-hospitalized patients.

Publication	Studied Outcome	Treated Group (Events)	Untreated/Placebo Group (Events)	Relative Risk/Hazard Ratio
[18]	Hospitalization or death	1039 (8)	1046 (66)	RR 0.101
[19]	All-cause ED visit	2547 (114)	2547 (138)	OR 0.818
	Hospitalization	2547 (15)	2547 (43)	OR 0.345
	Thirty-day mortality	2547 (0)	2547 (10)	-
	Composite of the above	2547 (125)	2547 (179)	OR 0.683
[20]	Hospitalization	98,060 (652)	914,850 (8285)	RR 0.742
	Death	98,060 (35)	914,850 (1480)	RR 0.269
[21]	Emergency room visit	1130 (82)	1130 (142)	RR 0.59
	Hospitalization	1130 (10)	1130 (23)	RR 0.40
	Death	1130 (0)	1130 (10)	RR 0.00
	Composite of the above	1130 (89)	1130 (144)	RR 0.55
[22]	Hospitalization	104,510 (628)	306,132 (5549)	aOR 0.35
[23]	Hospitalization (65+)	2484 (11)	40,337 (766)	aHR 0.27
	Hospitalization (65+, no previous immunity)	NA	NA	aHR 0.15
	Hospitalization (65+, previous immunity)	NA	NA	aHR 0.32
	Death (65+)	2484 (2)	40,337 (158)	aHR 0.21
	Hospitalization (40–64)	1418 (7)	65,015 (327)	aHR 0.74
	Hospitalization (40–64, no previous immunity)	NA	NA	aHR 0.23
	Hospitalization (40–64, previous immunity)	NA	NA	aHR 1.13
	Death (40–64)	1418 (1)	65,015 (16)	aHR 1.32
[24]	Hospitalization	99 (2)	103 (8)	aOR 0.16
[25]	Hospitalization or death	12,541 (69)	32,010 (310)	aRR 0.56
	Hospitalization or death (not fully vaccinated)	682 (NA)	3633 (NA)	aRR 0.19
	Hospitalization or death (vaccinated)	11,859 (NA)	28,377 (NA)	aRR 0.69
	Hospitalization	12,541 (NA)	32,010 (NA)	aRR 0.60
	Death	12,541 (NA)	32,010 (NA)	aRR 0.29
[26]	Hospitalization	198,927	500,921	HR 0.49
	Hospitalization (three doses mRNA vaccine)	NA	NA	HR 0.50
	Hospitalization (unvaccinated)	NA	NA	HR 0.50
[27]	Hospitalization or death	4737 (39)	175,614 (903)	HR 0.54
[28]	All-cause death	195 (11)	258 (28)	HR 0.4
	Twenty-eight-day mortality	195 (9)	258 (26)	OR 0.43
	Invasive ventilation	195 (12)	258 (17)	OR 0.93
	Non-invasive ventilation	195 (7)	258 (20)	OR 0.44
	ICU admission	195 (17)	258 (42)	OR 0.49
[29]	Death	890 (32)	890 (92)	HR 0.34
	Invasive mechanical ventilation	890 (6)	890 (6)	HR 0.97
	ICU admission	890 (0)	890 (1)	HR NA
	Needing oxygen therapy	890 (79)	890 (102)	HR 0.73
	Composite of the above	890 (101)	890 (173)	HR 0.57
[30]	ICU admission	28 (0)	64 (12)	Not provided
	Death	28 (1)	64 (17)	Not provided
[31]	Death	132 (5)	132 (8)	Not provided
[32]	Severe/critical illness or death	420,996 (1362)	1,515,959 (5229)	OR 0.568
	Severe/criticalillness or Death (60+)	377,531 (NA)	1,117,811 (NA)	OR 0.540
	Severe/critical illness or death (70+)	203,586 (NA)	481,326 (NA)	OR 0.537
	Severe/critical illness or death (80+)	81,736 (NA)	157,366 (NA)	OR 0.551
	Severe/critical illness or death (unvaccinated)	17,965 (NA)	57,829 (NA)	OR 0.392
	Severe/critical illness or death (vaccinated)	403,031 (NA)	1,458,130 (NA)	OR 0.628
	Death	420,996 (770)	1,515,959 (2321)	OR 0.689
[33]	Severe illness or death	623 (23)	196 (14)	aRR 0.49
	Death	623 (22)	196 (11)	aRR 0.62
[34]	Sixty-day mortality	280 (NA)	509 (NA)	aHR 0.71

## Data Availability

No new data were created or analyzed in this study. Data sharing is not applicable to this article.

## Supplementary Materials

The following supporting information can be downloaded at: https://www.mdpi.com/article/10.3390/arm92010009/s1, Table S1: Characteristics of the reviewed 17 studies. Table S2: Publications which were considered in our review. Table S3: Publications which were considered in our paper, but not in the review itself. Table S4. Publications, which were taken into consideration, but which did not meet our inclusion criteria and were hence not included in the review.
