# Supplementary material for: Clinical Effectiveness of Ritonavir-Boosted Nirmatrelvir—A Literature Review
Source: Adv Respir Med. 2024 Jan 18;92(1):66–76. doi: 10.3390/arm92010009 (PMC10801539; doi:10.3390/arm92010009)
Supplement: Supplementary file 1 [file arm-92-00009-s001.zip › arm-2760384-supplementary.pdf]

advances in respiratory medicine

MDPI

Supplementary Materials

Table S1. Characteristics of the reviewed 17 studies.

Publication	Type of Study	Inclusion Criteria	Exclusion Criteria	No. of participants	Geographic region	Study period
[1]	Retrospective study	18+, hospitalized		92	Saudi-Arabia	Prescription between January and December 2022
[2]	Observation, retrospective cohort study	40+, confirmed COVID-19 infection, considered high risk for severe disease	Treatment with molnupiravor, patients residing in long-term care facilities, patients hospitalized during the study period but before the positive SARS-CoV-2 test result	109,254	Israel	Jan 9, 2022 until March 31, 2022
[3]	Retrospective cohort study	18+, one or more risk factors for severe COVID-19 as per CDC guidelines (including 50+ years old), presence of underlying medical conditions associated with a conclusive higher risk of severe COVID-19	<18, patients hospitalized on or before the COVID-19 index date or date of treatment with nirmatrelvir/ritonavir, patients receiving nirmatrelvir/ritonavir before their index date, late beginning of treatment (later than 5 days after the patient's index date)	410,642	USA	Positive SARS-CoV-2 test or COVID-19 diagnosis between December 23, 2021 and December 31, 2022
[4]	Retrospective cohort study	18+, hospitalized and eGFR < 90ml/min (chronic kidney disease)	Hemodialyzed patients, peritoneal patients, pregnancy and breastfeeding, patients taking drugs such as amiodarone, propafenone and warfarin	1,279	China	April 7, 2022 and June 21, 2022
[5]	Retrospective controlled study	High-risk outpatients (patients with comorbidities		386	Italy	Diagnosis between February 1, 2022 and May 31, 2022

Publication	Type of Study	Inclusion Criteria	Exclusion Criteria	No. of participants	Geographic region	Study period
		and/or immune depression)				
[6]	Population-based cohort study	50+, nonhospitalized, no contraindications for nirmatrelvir plus ritonavir	PCR positive in the 90 days before the diagnosis, patients with an estimated glomerular filtration rate less than 30mL/min	44,551	USA	Diagnosis between Jan 1, 2022 and July 17, 2022
[7]	Retrospective cohort study	18-50 years old, vaccination, development of COVID-19 at least one month after vaccination	Treatment with other approved COVID-19 medications (monoclonal antibodies, convalescent plasma, molnupiravor)	5,094	USA	December 1, 2021 until July 30, 2022
[8]	Comparative retrospective cohort study	18+, vaccinated, development of COVID-19 at least one month after vaccination	Treatment with monoclonal antibodies, convalescent plasma, molnupiravir; receipt of nirmatrelvir/ritonavir more than five days after diagnosis; patients requiring initial hospitalization	2,260		Diagnosis between Dec 1, 2021 and Apr 18, 2022
[9]	phase 2-3, double-blind, randomized, placebo-controlled trial	18+, confirmed SARS-CoV-2 infection, symptom onset of no more than five days before randomization, have at least one sign or symptom of COVID-19 on the day of randomization and at least one characteristic or coexisting condition associated with	Previous vaccination or receipt of convalescent plasma, previous confirmed COVID-19 infection or hospitalization for COVID-19, and anticipated need for hospitalization within 48 hours of randomization	2,246	Participants from the United States, Bulgaria, South Africa, Brazil, India, Mexico, Ukraine, Turkey, Japan, Spain, Russia, Argentina, Colombia, Poland, South Korea, Hungary, Taiwan,	“Delta dominance”

Publication	Type of Study	Inclusion Criteria	Exclusion Criteria	No. of participants	Geographic region	Study period
		high risk of progression to severe COVID-19.			Malaysia, Czech Republic, Thailand and Puerto Rico	
[10]	Retrospective cohort study	18+, at least one risk factor for severe COVID-19	Contraindicated medical conditions, usage of one or more contraindicated medications, hospitalization within 3 days of index	1,029,910	USA	Positive test or diagnosis between December 2021 and February 2023
[11]	Retrospective cohort study	12+, prescription within 5 days of symptom onset, untreated patients who met the same eligibility criteria as in the nirmatrelvir/ritonavir guidelines (60+ OR 12+ (weighing at least 40kg) with underlying disease and/or immunosuppression, no supplemental oxygen)	Treatment wtith molnupiravir or remdesivir, < 12, missing data in basic epidemiological investigation and patients who did not meet the nirmatrelvir/ritonavir eligbility criteria	1,936,925	South Korea	Diagnosis between July 1 until November 30, 2022
[12]	Randomized controlled trial	18-90 years old, hospitalized with severe comorbidities, admission within 48 hours of positive PCR test	History of active liver disease, patients on dialysis or EGFR y45 mL/min/1.73m^2 within the past 6 months, HIV infection, history of allergy to nirmatrelvir/ritonavir	264	China	April 10, 2022 until May 19, 2022
[13]	Restrospective cohort study	18+, first-ever pos. COVID-19 test, at least one comorbidity or condition	Treatment with molnupiravor, late (> 5 days after positive test) beginning of	180,351	Israel	Diagnosis between Jan 1, 2022 and Feb 28, 2022

Publication	Type of Study	Inclusion Criteria	Exclusion Criteria	No. of participants	Geographic region	Study period
		associated with high risk for severe COVID-19 (including age 60+ years, body mass index (BMI) >= 30 kg/m^2, diabete, hypertension, cardiovascular disease, chronic liver disease, chronic lung disease, chronic kidney disease, neurological disease, immunosuppression, and melignancy)	treatment, use of medications that were contraindicated for use with Nirmatrelvir/Ritonavir, estimated glomerular filtration rate < 30 mL/minute/1.73m^2, dialysis, weight < 40kg, pregnancy			
[14]	Retrospective cohort study	Patients and workers at long-term care facitlies		2,241	South Korea	February 6, 2022 until April 2, 2022
[15]	Retrospective study	18+, outpatient encounter associated with the COVID-19 diagnosis, at least one previous face-to-face encounter in Cosmos during the 3 years preceding the diagnosis	Pregnancy, pharmacologic or medical contraindications to nirmatrelvir/ritonavir usage	699,848	USA	April until August 2022
[16]	Retrospective study	Severely and critically ill hospitalized patients	30 mL/min ≤ creatinine clearance < 60 mL/min, severe liver dysfunction (Child-Pugh C), known or suspected pregnancy, hypersensitivity to an nirmatrelvir/ritonavir, HIV infection, receiving Salmeterol, Rifampicin,	1,082	China	December 8, 2022 until February 9, 2023

Publication	Type of Study	Inclusion Criteria	Exclusion Criteria	No. of participants	Geographic region	Study period
			Tacrolimus, Sirolimus, Domperidone, Simvastatin, Rivaroxaban, Estazolam, Atorvastatin, Amiodarone, Propafenone, or Carbamazepine as usual medications prior to this hospitalization or any administration of drugs mentioned above within 72 h prior to assessment of eligibility			
[17]	Retrospective cohort study	Hospitalized, non-oxygen-dependent patients, hospital admission date within 3 days before or after COVID-19 diagnosis	< 18, admission to hospital more than 5 days after symptom onset, history of oral antiviral use before admission, requirement of supplemental oxygen on admission, drug-related contraindications to nirmatrelvir/ritonavir use, severe renal or severe liver impairment	1,780	Hong Kong	Diagnosis between Feb 26, 2022 and Apr 26, 2022

This supplementary material contains three tables: The first table contains the preprints and papers, which met the inclusion criteria and were considered in our review. The second table contains the preprints and papers, which were included in our study, but not the review itself and the final table includes all papers and preprints, whose title and abstracts were scanned, but which did not meet the inclusion criteria.

Used

Table S2. Publications which were considered in our review.

Author(s)	Title	Type of publication & journal
[1]	Paxlovid for Treating COVID-19 Patients: A Case- Control Study From Two Hospitals in the Eastern Province of Saudi Arabia	Published in *Cureus*
[2]	Nirmatrelvir Use and Severe Covid-19 Outcomes during the Omicron Surge	Published in *The New England Journal of Medicine*
[3]	Effect of Nirmatrelvir/Ritonavir (Paxlovid) on Hospitalization among Adults with COVID-19: an EHR-based Target Trial Emulation from N3C	Preprint
[4]	Paxlovid for hospitalized COVID-19 patients with chronic kidney disease	Published in *Antiviral Research*
[5]	Molnupiravir, Nirmatrelvir/Ritonavir, or Sotrovimab for High-Risk COVID-19 Patients Infected by the Omicron Variant: Hospitalization, Mortality, and Time until Negative Swab Test in Real Life	Published in *pharmaceuticals*
[6]	Nirmatrelvir Plus Ritonavir for Early COVID-19 in a Large U.S. Health System	Published in *Annals of Internal Medicine*
[7]	Oral Nirmatrelvir and Ritonavir for Coronavirus Disease 2019 in Vaccinated, Nonhospitalized Adults Aged 18–50 Years	Published in *Clinical Infectious Diseases*
[8]	Oral Nirmatrelvir and Ritonavir in Non-hospitalized Vaccinated Patients with Covid-19	Published in *Clinical Infectious Diseases*

[9]	Oral Nirmatrelvir for High-Risk, Nonhospitalized Adults with Covid-19	Published in *New England Journal of Medicine*
[10]	Paxlovid (nirmatrelvir/ritonavir) effectiveness against hospitalization and death in N3C: A target trial emulation study	Preprint
[11]	Effectiveness of Paxlovid, an Oral Antiviral Drug, Against the Omicron BA.5 Variant in Korea: Severe Progression and Death Between July and November 2022	Published in *Journal of Korean Medical Science*
[12]	Efficacy and safety of Paxlovid in severe adult patients with SARS-Cov-2 infection: a multicenter randomized controlled study	Published in *The Lancet Regional Health - Western Pacific*
[13]	Effectiveness of Nirmatrelvir/Ritonavir in Reducing Severe Coronavirus Disease 2019 and Mortality in High-Risk Patients	Published in *Clinical Infectious Diseases*
[14]	The effectiveness of Paxlovid treatment in long-term care facilities in South Korea during the outbreak of the Omicron variant of SARS-CoV-2	Published in Osong Public Health and Research Perspectives
[15]	Paxlovid associated with decreased hospitalization rate among adults with COVID-19 — United States, April–September 2022	Published in *American Journal of Transplantation*
[16]	Composite Interventions on Outcomes of Severely and Critically Ill Patients with COVID-19 in Shanghai, China	Published in *Microorganisms*
[17]	Real-world effectiveness of early molnupiravir and nirmatrelvir/ritonavir among hospitalized, non-oxygen-	Preprint

	dependent COVID-19 patients on admission during Hong Kong's Omicron BA.2 wave: an observational study	

Used, but not in review

Table S3. Publications which were considered in our paper, but not in the review itself.

Author(s)	Summary	Used in section
[18]	Examination (clinically, virologically and immunologically) of seven patients who experienced rebound symptoms. Six experienced rebound symptoms after taking Nirmatrelvir/Ritonavir, the final patient did not receive the treatment.	Discussion
[19]	Short sections on who is eligible for treatment with Nirmatrelvir/Ritonavir, how does Nirmatrelvir/Ritonavir work, what peer reviewed evidence is there for Nirmatrelvir/Ritonavir, which countries are using Nirmatrelvir/Ritonavir, how much does Nirmatrelvir/Ritonavir cost, what is the potential for this drug.	Nirmatrelvir/Ritonavir
[20]	Argue that Nirmatrelvir/Ritonavir might harm transplant patients as there is a chance of medication interaction and that the ritonavir component of Nirmatrelvir/Ritonavir might pose a safety risk for these patients	Discussion
[21]	Enrollment of around 260 patients of which	Methods

Author(s)	Summary	Used in section
	approximately half were treated with molnupiravir, while the other half was treated with Nirmatrelvir/Ritonavir. Recorded hospitalization and deaths during a 14-day follow-up period. As no (untreated) control group is provided, this work is not included in the review.	
[22]	Discussion of if and how rebound symptoms after taking Nirmatrelvir/Ritonavir differ between Omicron BA.5 and Omicron BA.2.12.1 infected patients	Discussion
[23]	Review which summarized the effects of ritonavir on drug disposition and debates what influences the probability of drug-drug interaction when taking Nirmatrelvir/Ritonavir.	Discussion
[24]	Discusses side-effects of Nirmatrelvir/Ritonavir in a single kidney transplant patient, whos treatment with Nirmatrelvir/Ritonavir had to be interrupted and who suffered acute kidney injury from taking the drug	Discussion
[25]	No abstract available on Pubmed, published under "Medical News & Perspectives", discussing rebound effects potentially associated with Nirmatrelvir/Ritonavir treatment.	Discussion
[26]	Assessment of the in vitro antiviral effect of GS-441524, remdesivir, EIDD-1931,	Discussion

Author(s)	Summary	Used in section
	molnupiravir and nirmatrelvir against the various SARS- CoV-2 VOCs, including Omicron.	
[27]	Review of efficacy and safety of the three antiviral treatments molnupiravir, fluvoxamine and Nirmatrelvir/Ritonavir. Published, when only Nirmatrelvir/Ritonavir's clinical results were available.	Introduction

Unused Table

Table S4. Publications, which were taken into consideration, but which did not meet our inclusion criteria and were hence not included in the review.

Author(s)	Summary	Reason for Exclusion
[28]	Discussing Thymoquinone and other natural products as possible treatments for COVID-19.	Does not meet the inclusion criteria.
[29]	Case discussion of two patients who were prescribed Nirmatrelvir/Ritonavir and experiences a rebound of COVID-19	Does not meet the inclusion criteria.
[30]	Nirmatrelvir/Ritonavir binds non-covalently at regions other than the catalytic sites with energies stronger than reported, namely to mMpro.	Does not meet the inclusion criteria.
[31]	Review which presents the efficacy of repurposed drugs for COVID-19. Discussion of clinical trials, combination therapies and novel methods followed for treatment. Written when Nirmatrelvir/Ritonavir was undergoing Phase III studies.	Does not meet the inclusion criteria.
[32]	Debating evidence from pivotal	Does not meet the inclusion

Author(s)	Summary	Reason for Exclusion
	trials that led to the approval of effective COVID-19 therapeutics and categorizing them as effective outpatient and inpatient management strategies.	criteria.
[33]	Description of drugs that contraindicated and/or should (not) be used carefully when also taking Nirmatrelvir/Ritonavir based on fact sheets by the Spanish Agency for Medicines and Health Products.	Does not meet the inclusion criteria.
[34]	Proposition of a hybrid multiscale mathematical approach to assess Nirmatrelvir/Ritonavir.	Does not meet the inclusion criteria.
[35]	Usage of techniques of structure modeling, in silico docking and pharmacokinetics prediction to test compounds from algae for their ability to inhibit SARS-CoV-2's protease Mpro.	Does not meet the inclusion criteria.
[36]	No abstract available on pubmed.	Abstract unavailable.
[37]	Discussion of the case of a male veteran reporting rebound symptoms after Nirmatrelvir/Ritonavir treatment. The patient was found to be hypoxic with pulmonary emboli.	Does not meet the inclusion criteria.
[38]	No abstract available on pubmed.	Abstract unavailable.
[39]	No abstract available on pubmed.	Abstract unavailable.
[40]	No abstract available on pubmed.	Abstract unavailable.

Author(s)	Summary	Reason for Exclusion
[41]	The English abstract solely states that Nirmatrelvir/Ritonavir is an available treatment for COVID-19 infections, administered orally and to people at high risk for severe disease.	Only the abstract was available in English.
[42]	No abstract available on pubmed.	Abstract unavailable.
[43]	Written when Nirmatrelvir/Ritonavir was still under development, embryo-fetal development studies in rats and rabbits to assess clinically relevant risks when prescribing Nirmatrelvir/Ritonavir to males and females in reproductive age.	Does not meet the inclusion criteria.
[44]	Aim to critically assess benefits and shortcomings of using molecular models for drug repurposing when trying to develop effective COVID-19 treatments..	Does not meet the inclusion criteria.
[45]	No abstract available on pubmed.	Abstract unavailable.
[46]	Discussing panax ginseng's (a medical plant) ability to control cytokine storm in COVID-19	Does not meet the inclusion criteria.
[47]	Identification of patients potentially eligible for treatment with Nirmatrelvir/Ritonavir in the UK. In consequence, assessment of the coverage of new treatments among these patients with the conclusion that there were variants in coverage between key clinical,	Does not meet the inclusion criteria.

Author(s)	Summary	Reason for Exclusion
	geographic and demographic groups.	
[48]	No abstract available on pubmed.	Abstract unavailable.
[49]	No abstract available on pubmed.	Abstract unavailable.
[50]	For 36 mRNA-vaccinated and Omicron-infected individuals viral kinetics were measured. 11 of the 36 were treated with Nirmatrelvir/Ritonavir and treatment was associated with larger incidence of viral rebound.	Does not meet the inclusion criteria.
[51]	Report of simulation of H172Y mutation on Mpro's structure leading to decreased structural stability and binding affinity.	Does not meet the inclusion criteria.
[52]	Evaluation of the incidence of viral and symptom rebound in untreated mild to moderate COVID-19 outpatients.	Does not meet the inclusion criteria.
[53]	Review presenting the progress in clinical trials concerning the effectiveness of treatments for COVID-19. Written when solely the results from Nirmatrelvir/Ritonavir's clinical trials were available.	Does not meet the inclusion criteria.
[54]	No abstract available on pubmed.	Abstract unavailable.
[55]	Presenting the preclinical disposition, metabolism and potential drug-drug interaction of nirmatrelvir.	Does not meet the inclusion criteria.
[56]	Discussion of levels of total cholesterol, LDL-C, HDL-C, and apolipoprotein B and A-I levels in patients with COVID-19 infections.	Does not meet the inclusion criteria.

Author(s)	Summary	Reason for Exclusion
[57]	Discussing the potential of Yindan Jiedu granules as a treatment for COVID-19. Comparison of Yindan Jiedu granules and Nirmatrelvir/Ritonavir when treating COVID-19 patients.	Does not meet the inclusion criteria.
[58]	Present how Pfizer has successfully improved its research and development (R&D) productivity between 2010 and 2020.	Does not meet the inclusion criteria.
[59]	Presentation of literature on monotherapy use of Nirmatrelvir/Ritonavir and monotherapy use of remdesivir and discussion of the hypothesis of using nirmatrelvir and remdesivir to increase efficacy of COVID-19 treatment.	Does not meet the inclusion criteria.
[60]	No abstract available on pubmed.	Abstract unavailable.
[61]	Provides a review of currently available COVID-19 treatments. As this was published in December 2021, it only include the clinical trials for Nirmatrelvir/Ritonavir.	Does not meet the inclusion criteria.
[62]	Data from December 2021 until May 2022 were analyzed to describe oral antiviral treatment prescription dispensing overall and by week, stratified by zip code and social vulnerability.	Does not meet the inclusion criteria.
[63]	Evaluation of in vitro potency of normatrelvir against the Mpro of currently circulating (and previous) variants of	Does not meet the inclusion criteria.

Author(s)	Summary	Reason for Exclusion
	concern. Their in vitro data suggests that Nirmatrelvir/Ritonavir has the ability to inhibit SARS-CoV-2 replication, even vor VOCs like Omicron.	
[64]	No abstract available on pubmed.	Abstract unavailable.
[65]	Retrospective analysis of benefits and limitations of (previous) treatments for COVID-19. Calls Nirmatrelvir/Ritonavir a promising treatment, but only presents clinical results.	Does not meet the inclusion criteria.
[66]	People with cystic fibrosis (CF) are at risk for drug-drug interaction when being treated with Nirmatrelvir/Ritonavir. Simulation of coadministration of elexacaftor-tezacaftor-ivacaftor and Nirmatrelvir/Ritonavir to examine these potential interactions.	Does not meet the inclusion criteria.
[67]	Case report of a severe aplastic anemia child who was successfully treated with Nirmatrelvir/Ritonavir.	Does not meet the inclusion criteria.
[68]	Written before Nirmatrelvir/Ritonavir was authorized and before clinical results were available. Hence, discussing the promising antiviral effect of nirmateelvir, while also naming (then) unresolved concerns.	Does not meet the inclusion criteria.
[69]	Evaluation of drugs authorized in the US for treating COVID-19. For Nirmatrelvir/Ritonavir's	Does not meet the inclusion criteria.

Author(s)	Summary	Reason for Exclusion
	effectiveness, solely the clinical results are discussed.	
[70]	Discussing medical management (but excluding intensive care management) of COVID-19.	Article only available in French.
[71]	Review to present the potential of nirmatrelvir when treating COVID-19, discussing its history of rational design, its target selectivity, synthesis and drug resistance.	Does not meet the inclusion criteria.
[72]	News article introducing the most important Nirmatrelvir/Ritonavir-related information for the general public and patients.	Does not meet the inclusion criteria.
[73]	No abstract available on pubmed.	Abstract unavailable.
[74]	No abstract available on pubmed.	Abstract unavailable.
[75]	Examination of rates and relative risks of COVID-19 rebound in patients treated with Molnupiravir or Nirmatrelvir/Ritonavir during January-June 2022	Does not meet the inclusion criteria.
[76]	Summary of the milestones during the development of normatrelvir plus ritonavir leading to its first authorizations and approval for the treatment of COVID-19	
[77]	No abstract available on pubmed.	Abstract unavailable.
[78]	No abstract available on pubmed.	Abstract unavailable.
[79]	Discussion of potential nirmatrelvir escape mutations from emerging variants of	Does not meet the inclusion criteria.

Author(s)	Summary	Reason for Exclusion
	SARS-CoV-2 and exploration of the mutational landscape of Mpro.	
[80]	Discussion of drug interactions of Nirmatrelvir/Ritonavir and immunosuppressant drugs and provision of general recommendations for therapeutic drug monitoring when co-administering the two.	Does not meet the inclusion criteria.
[81]	Providing recommendations on behalf of the national French society of pharmacology for possible drug-drug interactions between Nirmatrelvir/Ritonavir and other commonly used drugs.	Does not meet the inclusion criteria.
[82]	No abstract available on pubmed.	Abstract unavailable.
[83]	Introduction of a potential future treatment, the orally available ribonucleoside analog 4′-fluorouridine (4′-FIU). Furthermore, reviewing currently approved and emerging medicines against COVID-19.	Does not meet the inclusion criteria.
[84]	Discussing statin use in the context of COVID-19, coming to no evidence suggesting interference between statins and COVID-19 vaccines. But, simultaneous stantis and Nirmatrelvir/Ritonavir administration may increase statin exposure and the risk of adverse effects.	Does not meet the inclusion criteria.
[85]	Development of an efficient LC-MS/MS method for simultaneously determining	Does not meet the inclusion criteria.

Author(s)	Summary	Reason for Exclusion
	nirmatrelvir and ritonavir in human plasma.	
[86]	Show that Apilimod and other PIKfyve inhibitors worsen disease in a COVID-19 murine model when given prophylactically or therapeutically. Abstract solely mentions Nirmatrelvir/Ritonavir as an available treatment.	Does not meet the inclusion criteria.
[87]	Multicentre cohort study describing clinical characteristics, and assessing risk and protective factors for geriatric Omicron severe infections.	
[88]	Discussing herbal extracts as potential treatments for COVID-19. Solely mentions that molnupiravir and Nirmatrelvir/Ritonavir are not widely available.	Does not meet the inclusion criteria.
[89]	News article in BMJ, published shortly after the analysis of the phase II-III data was published. Hence, discussing and reporting the results of this analysis.	Does not meet the inclusion criteria.
[90]	Consideration of electronic health record from a large integrated health care system in California to analyze and quantify hospital admissions and emergency department encounters related to COVID-19 during the 5-15 days after receiving Nirmatrelvir/Ritonavir	Does not meet the inclusion criteria.

Author(s)	Summary	Reason for Exclusion
	treatment	
[91]	No abstract available, article in French.	
[92]	Report the discovery and description of nirmatrelvir. Comparison of effectiveness of molnupiravir and normatrelvir). Written while Nirmatrelvir/Ritonavir was under study in phase III of the clinical trial.	Does not meet the inclusion criteria.
[93]	Praxis-relevant recommendations for the usage and prescription of Paxlobid.	Does not meet the inclusion criteria.
[94]	No abstract available on pubmed.	
[95]	News article published in EMBO Molecular Medicine, discussing [96]	Does not meet the inclusion criteria.
[97]	Compilation of a list of drugs and their potentially relevant interactions when being administered simultaneously with Nirmatrelvir/Ritonavir as well as a list of commonly prescribed drugs for which no such interactions exist.	Does not meet the inclusion criteria.
[98]	Discussion of how ongoing virus evolution has the potential to yield variants with resistance to clinical protease inhibitors (like Nirmatrelvir/Ritonavir).	Does not meet the inclusion criteria.
[99]	Published when solely Nirmatrelvir/Ritonavir's clinical results were available. Hence, only these are discussed.	Does not meet the inclusion criteria.
[100]	Review of normatrelvir's binding to SARS-CoV-2's Mpro	Does not meet the inclusion criteria.

Author(s)	Summary	Reason for Exclusion
	and its potential inefficacy when novel mutations arise.	
[101]	Presentation of current antiviral drugs for COVID-19 as well as potential future directions.	Does not meet the inclusion criteria.
[102]	Review of literature on the drug design landscape of SARS-CoV-2 Mpro inhibitors. Mentions nirmatrelvir as one such inhibitor.	Does not meet the inclusion criteria.
[103]	Development of a non-pathogenic system in which yeast growth is a proxy for Mpro activity. Consequently, mutants which exhibit drug sensitivity and altered enzymatic activity can quickly be identified.	Does not meet the inclusion criteria
[104]	Discussion of review of risk factors for severe covid and Pre-exposure prophylaxis (PrEP) strategies against SARS-CoV-2. Additionally, presentation of potential SARS-CoV-2 PrEP drugs	Does not meet the inclusion criteria.
[105]	Published in late 2021, reporting the current status of oral treatments for COVID-19	Does not meet the inclusion criteria.
[106]	Analysis of newly reported bat coronaviruses with regard to the similarities and differences between their 3CL protease and SARS-CoV-2.	Does not meet the inclusion criteria
[107]	Analysis of the structural features of the Spike protein and the Mpro of the SARS-CoV-2 variant XE and the closely related variants XD and XF.	Does not meet the inclusion criteria.
[108]	Comparison and changes in the epidemiology, clinical profile,	Does not meet the inclusion criteria.

Author(s)	Summary	Reason for Exclusion
	therapeutics and public health measures for the COVID-19 pandemic in the Asia Pacific region. Abstract mentions recent introduction of Nirmatrelvir/Ritonavir.	
[109]	Report of 4 cases from a post-COVID cohort study who received nirmatrelvir as part of clinical care and who experiences different outcomes.	Does not meet the inclusion criteria.
[110]	Published in late 2021, when the federal government rationed Nirmatrelvir/Ritonavir doses. Hence, this paper identifies relevant ethical principles and priority groups for access to Nirmatrelvir/Ritonavir.	Does not meet the inclusion criteria.
[111]	No abstract available on pubmed.	
[112]	No abstract available on pubmed.	
[113]	Examine modifications of a promising peptide-based inhibitor of the spike protein, LCB3, against common mutations in the target protein. In consequence, LCB3 retains its efficacy against the spike protein.	Does not meet the inclusion criteria.
[114]	Discussing Nirmatrelvir/Ritonavir's efficacy after publication of clinical results. Article is in Spanish.	Does not meet the inclusion criteria.
[115]	Discussion of a new class of sartan-like arterial antihypertensive drugs (referred to as "bisartans") as treatment for COVID-19.	Does not meet the inclusion criteria.

Author(s)	Summary	Reason for Exclusion
	Abstract notes that bisartans do not inhibit SARS-CoV-2 infection in bioassays as effectively as Nirmatrelvir/Ritonavir.	
[116]	No abstract available on pubmed.	
[117]	Discussion of risk of drug interactions between treatments for COVID-19 and drugs used in treating comorbid conditions like diabetes or cardiovascular illness.	Does not meet the inclusion criteria.
[118]	Summarizes diagnostic and therapeutic management of COVID-19 for outpatients. Abstract names Nirmatrelvir/Ritonavir was the preferred treatment for mild cases with high risk of disease progression.	Does not meet the inclusion criteria.
[119]	Assessing currently available COVID-19 drugs for their potential toxicological effects and mechanisms.	Does not meet the inclusion criteria.
[120]	Describes clinical experience with 25 organ transplant recipients who were prescribed Nirmatrelvir/Ritonavir. Their results suggest that clinically significant interaction between Nirmatrelvir/Ritonavir and immunosuppressive agents can be reasonably managed with a standardized dosing protocol	Does not meet the inclusion criteria.
[121]	No abstract available on pubmed.	
[122]	Review of molnupiravir and Nirmatrelvir/Ritonavir with	Does not meet the inclusion criteria

Author(s)	Summary	Reason for Exclusion
	regard to their mechanisms of action, their antiviral activity, pharmacokinetics, drug interactions and clinical experience including trials, adverse events, recommended indications and formulary considerations.	
[123]	Providing vivo safety assessments of Nirmatrelvir/Ritonavir by studies in rats and monkeys.	Does not meet the inclusion criteria.
[124]	Simulation of molnupiravir treatment to judge effectiveness of antiviral therapy in highly-transmissible variants.	Does not meet the inclusion criteria.
[125]	No abstract available on pubmed.	
[126]	Discussing the intrinsic stability of Nirmatrelvir and the degrading products formed under forced conditions.	Does not meet the inclusion criteria.
[127]	No abstract available on pubmed.	
[128]	Introduce the ability of the combination of pamapimod and pioglitazone to inhibit SARS-CoV-2 replication in vitro. Hence, this combination is a potential treatment of COVID-19 and is evaluated (at the time of writing) in a phase II clinical study.	Does not meet the inclusion criteria.
[129]	Provides practical clinical guidelines for using molnupiravor in COVID-19 patients.	Does not meet the inclusion criteria.
[130]	Discussing contraceptive care in times of the Pandemic. Abstract mentions that	Does not meet the inclusion criteria.

Author(s)	Summary	Reason for Exclusion
	combined hormonal contraceptive users who take Nirmatrelvir/Ritonavir should consider an additional contraceptive method for the duration of Nirmatrelvir/Ritonavir treatment.	
[131]	No abstract available on pubmed.	
[132]	Development and validation of a method to quantify almonertinib in rat plasma to study the effects of Nirmatrelvir/Ritonavir on the pharmacokinetics of almonertinib in rats.	Does not meet the inclusion criteria.
[133]	No abstract available on pubmed.	
[134]	Presenting the role of endothelial and vascular components as major targets for COVID-19-induced tissue injury, spreading to various organs and injury healing as well as current COVID-19 treatments.	Does not meet the inclusion criteria.
[135]	Israelian study to determine the number of patients who could be included in a prospective Real World Evidence Study to study Nirmatrelvir/Ritonavir's effect on patients' outcomes as well as assessment of comparability between patients who received the treatment and patients who did not.	
[136]	Discussing the important role of the signal transducer and	Does not meet the inclusion criteria.

Author(s)	Summary	Reason for Exclusion
	activator of transcription (STAT) 1 protein in antiviral immune response and how viruses like Ebola and SARS-CoV-2 have developed the ability to inhibit this transcription factor.	
[137]	No abstract available on pubmed.	
[138]	No abstract available on pubmed.	
[139]	No abstract available on pubmed.	
[140]	Review of the indications of immunosuppresants and immunomodulators and guidance on which mild to moderate COVID-19 patients might benefit from their use in dermatology.	Does not meet the inclusion criteria.
[141]	Review which provides an overview regarding the molecular profile of the Omicron variant, as well as its transmissibility and the remaining vaccine effectiveness. Mentioned molnupiravir and Nirmatrelvir/Ritonavir as oral treatments of COVID-19.	Does not meet the inclusion criteria.
[142]	Written when the Omicron variant hat just emerged and molnupiravir and Nirmatrelvir/Ritonavir had only undergone clinical study. Briefly reviewing the impact of these two new oral antivirals.	Does not meet the inclusion criteria.
[143]	Discussion of drug interactions when simultaneously taking	Does not meet the inclusion criteria.

Author(s)	Summary	Reason for Exclusion
	Nirmatrelvir/Ritonavir and cholesterol-lowering drugs.	
[144]	Summary of the current progress in the structural biology of SARS-CoV-2 as well as presentation of structure-based design of Nirmatrelvir/Ritonavir, molnupiravor and VV116 to emphasize the importance of structure in drug development for COVID-19	Does not meet the inclusion criteria.
[145]	No abstract available on pubmed.	
[146]	Recruitment of 5 pediatric cases with underlying disease who were treated with Nirmatrelvir/Ritonavir as well as 30 age-matched patients with underlying disease who were not treated with Nirmatrelvir/Ritonavir as controls. Assessment of efficacy and safety of Nirmatrelvir/Ritonavir as well as inter-group comparisons.	Does not meet the inclusion criteria.
[147]	From evolutionary and structural standpoints possible mutations in Mpro leading to evasion of nirmatrelvir are discussed.	Does not meet the inclusion criteria.
[148]	Case report of a 14 year old female kidney transplant, whose COVID-19 infection was treated with Nirmatrelvir/Ritonavir.	Does not meet the inclusion criteria.
[149]	Cystic fibrosis patients are at increased risk for drug-drug interaction when taking Nirmatrelvir/Ritonavir. Here,	Does not meet the inclusion criteria.

Author(s)	Summary	Reason for Exclusion
	these interactions are explored using a physiologically- based pharmacokinetic modeling approach.	
[150]	Letter to the editor discussing the potential of Nirmatrelvir/Ritonavir to treat COVID-19	Does not meet the inclusion criteria.
[151]	Review presenting an update on fluorinated COVID-19 drugs. Here, current knowledge of these drugs' molecular design, metabolism and pharmacokinetics as well as mechanism of action, is provided.	Does not meet the inclusion criteria.
[152]	Review which aims to provide a compact and updated summary of pediatric COVID-19 diagnosis and management. Abstract mentions that in patients at increased risk of progression, Nirmatrelvir/Ritonavir (among other treatments) should be considered.	Does not meet the inclusion criteria.
[153]	No abstract available on pubmed.	Abstract unavailable.
[154]	Study of some of the key human transporters interacting with Molnupiravir and Nirmatrelvir.	Does not meet the inclusion criteria.
[155]	No abstract available on pubmed.	Abstract unavailable.
[156]	Exploration of several reactive warheads in the design of Mpro inhibitors, as SARS-CoV-2 main protease (Mpro) is a validated antiviral drug target of nirmatrelvir.	Does not meet the inclusion criteria.

Author(s)	Summary	Reason for Exclusion
[157]	Presentation of the case of a small bowel transplant patient who had a COVID-19 infection by discussing the management experience and reviewing the relevant literature.	Does not meet the inclusion criteria.
[158]	Listing of etiopathogenic theories of long coronavirus disease and discussion of currently existing main lines of treatment.	Does not meet the inclusion criteria.
[159]	Discussion of possible means to prevent Long COVID, including vaccination, orally effective antiviral agents, and presentation of ongoing clinical trials on dietary supplements or drugs with different mechanisms of action.	Does not meet the inclusion criteria.
[160]	Exploration of the cause for prolonged viral shedding time to establish increased age, comorbidities, incomplete vaccination, severe or critical infections, and delayed Paxlovid treatment as the risk factors.	Does not meet the inclusion criteria.
[161]	Suggestion of structural modification of nirmatrelvir to fight the drug resistance resulting from the appearance of many novel COVID-19 variants.	Does not meet the inclusion criteria.
[162]	No abstract available on pubmed.	Abstract unavailable.
[163]	Report a case of simultaneous mixed-type autoimmune hemolytic anemia with immune thrombocytopenia which followed after a severe infection	Does not meet the inclusion criteria.

Author(s)	Summary	Reason for Exclusion
	with COVID-19, treatment with Paxlovid and a subsequent Rhinovirus infection.	
[164]	Discuss the speed of the development of Nirmatrelvir.	Does not meet the inclusion criteria.
[165]	Application of a parsimonious mathematical COVID-19 immunity model to explore the effect of nirmatrelvir/ritonavir in (un)vaccinated patients and potentially occurring viral rebound.	Does not meet the inclusion criteria.
[166]	Case report of a heart-transplant patient to explore the drug-drug interaction between nirmatrelvir/ritonavir and Tacrolimus.	Does not meet the inclusion criteria.
[167]	Review the development of small molecule anti-SARS-CoV-2 drugs in China.	Does not meet the inclusion criteria.
[168]	Prediction of the length of stay (LoS) for hospitalized COVID-19 patients and analysis of cohorts of risk factors reducing or prolonging LoS.	Does not meet the inclusion criteria.
[169]	No abstract available on pubmed.	Abstract unavailable.
[170]	**Explore clinical efficacy of Paxlovid in children with hematological diseases.**	Does not meet the inclusion criteria.
[171]	Modeling study exploring the public health impact of bivalent vaccines and nirmatrelvir-ritonavir against COVID-19 and coming to the inclusion and coming to the conclusion that the most efficient strategy for both is to target the group of	Does not meet the inclusion criteria.

Author(s)	Summary	Reason for Exclusion
	people 75 and older.	
[172]	Exploration of risk factors for Omicron-associated pneumonia.	Does not meet the inclusion criteria.
[173]	No abstract available on pubmed.	Abstract unavailable.
[174]	Investigation of potential herb–drug interactions between traditional Chinese medicine and COVID-19 treatments.	Does not meet the inclusion criteria.
[175]	Investigation of the outcomes of Covid-19 infection in patients with Hairy cell leukemia.	Does not meet the inclusion criteria.
[176]	Assessment of the cost-effectiveness of Paxlovid in reducing severe COVID-19 and mortality in China to come to the conclusion that Paxlovid's only cost-effective in patients 80 and older (regardless of vaccination status).	Does not meet the inclusion criteria.
[177]	Modeling study to explain COVID-19 rebound after nirmatrelvir/ritonavir treatment.	Does not meet the inclusion criteria.
[178]	Discovery of novel bicyclic[3.3.0]proline peptidyl α-ketoamides as potent 3CL-protease inhibitors for SARS-CoV-2.	Does not meet the inclusion criteria.
[179]	Case study of a patient exhibiting petechial rash found to be seronegative IgA vasculitis presenting concurrently with dyspnea subsequently to being infected with COVID-19.	Does not meet the inclusion criteria.
[180]	Screening of a library of microbial metabolites to	Does not meet the inclusion criteria.

Author(s)	Summary	Reason for Exclusion
	discover new SARS-CoV-2 inhibitors to complement and extend existing COVID-19 drugs.	
[181]	Investigation of potential herb–drug interactions in low- and middle-income countries, in which access to Paxlovid has recently increased.	Does not meet the inclusion criteria.
[182]	No abstract available on pubmed.	Abstract unavailable.
[183]	Case study of a patient, who was treated with Paxlovid and continued to take Ranolazine, resulting in a drug-drug interaction and an emergency room visit.	Does not meet the inclusion criteria.
[184]	Introduction of novel Peptidomimetics for the treatment of COVID-19.	Does not meet the inclusion criteria.
[185]	Investigate the immunity acquired through BA.2 and BA.2.12.1 infections and come to the conclusion that these infections fail to protect against the BA.4/BA.5 variant.	Does not meet the inclusion criteria.
[186]	Present the case of a patient who suffered from acute pancreatitis after taking Paxlovid.	Does not meet the inclusion criteria.
[187]	Present the case of an elderly patient who, after taking Paxlovid, experienced medication interactions with benzodiazepines and narcotics leading to altered mental status.	Does not meet the inclusion criteria.
[188]	Present four cases who suffered from a rebound after taking Paxlovid and progressed to severe COVID-19.	Does not meet the inclusion criteria.

Author(s)	Summary	Reason for Exclusion
[189]	Literature review to examine if nirmatrelvir/ritonavir can be used for solid organ transplant recipients suffering from COVID-19.	Does not meet the inclusion criteria.
[190]	Perform a national survey on the nirmatrelvir/ritonavir prescribing processes and access barriers.	Does not meet the inclusion criteria.
[191]	Explore possible adverse cutaneous reactions resulting from nirmatrelvir/ritonavir intake.	Does not meet the inclusion criteria.
[192]	No abstract available on pubmed.	Abstract unavailable.
[193]	No abstract available on pubmed.	Abstract unavailable.
[194]	Present VV116, a chemically-modified version of the antiviral remdesivir, as a potential treatment for COVID-19.	Does not meet the inclusion criteria.
[195]	No abstract available on pubmed.	Abstract unavailable.
[196]	Discuss the potential of Paxlovid for treating mild COVID-19 cases of pregnant and/or lactating patients.	Does not meet the inclusion criteria.
[197]	Usage of deep learning to predict potential drug-drug interactions between Paxlovid and more than 2,000 prescription drugs.	Does not meet the inclusion criteria.
[198]	Explore resistance profiles of nirmatrelvir, ensitrelvir, and FB2001 to find that the next-generation drugs have the potential to be effective against nirmatrelvir-resistant variants.	Does not meet the inclusion criteria.
[199]	Present experience gained from	Does not meet the inclusion

Author(s)	Summary	Reason for Exclusion
	the COVID-19 pandemic and strategies for facing present and future pandemics.	criteria.
[200]	Review to present in-depth information on new molecules and repurposed drugs for the treatment of COVID-19.	Does not meet the inclusion criteria.
[201]	Discuss how administration of Paxlovid for the treatment of COVID-19 is feasible for solid organ transplant recipients with medical management.	Does not meet the inclusion criteria.
[202]	Case study of a patient who suffered from tacrolimus toxicity as a result of drug-drug interaction between Paxlovid and their home medications.	Does not meet the inclusion criteria.
[203]	Exploration of effectiveness of Favipiravir monotherapy in the treatment of COVID-19.	Does not meet the inclusion criteria.
[204]	Usage of molecular docking analysis to discover Nirmatrelvir's antiviral potency against different coranaviruses.	Does not meet the inclusion criteria.
[205]	Explore vaccination and treatment options for COVID-19 for lactating patients.	Does not meet the inclusion criteria.
[206]	No abstract available on pubmed.	Abstract unavailable.
[207]	Explore nirmatrelvir/ritonavir-associated reports of dysgeusia.	Does not meet the inclusion criteria.
[208]	Discuss potential use and dosing of nirmatrelvir/ritonavir for patients with chronic kidney disease.	Does not meet the inclusion criteria.
[209]	Explore factors associated with prolonged COVID-19 viral shedding in elderly Chinese patients.	Does not meet the inclusion criteria.

Author(s)	Summary	Reason for Exclusion
[210]	Case report of a patient who tested positive again for the Omicron BA.5 variant after being treated with nirmatrelvir/ritonavir.	Does not meet the inclusion criteria.
[211]	No abstract available on pubmed.	Abstract unavailable.
[212]	No abstract available on pubmed.	Abstract unavailable.
[121]	No abstract available on pubmed.	Abstract unavailable.
[213]	Demonstration of a 7-step, 3-pot synthesis of the antiviral nirmatrelvir, arriving at the targeted drug in 70% overall yield.	Does not meet the inclusion criteria.
[214]	Summary of the currently available data on ritonavir and nirmatrelvir during pregnancy as well as review of recommendations of major societies worldwide regarding Paxlovid use during pregnancy.	Does not meet the inclusion criteria.
[215]	Analysis of Google search queries to explore public interest in COVID-19 antivirals. Does not meet inclusion criteria.	Does not meet the inclusion criteria.
[216]	Design of mutations to impair binding of nirmatrelvir and demonstrate nirmatrelvir resistance in the COVID-19 BA.1 variant and WA1 replicons.	Does not meet the inclusion criteria.
[217]	Report the case of a patient who developed symptomatic bradycardia, syncopal episodes, and sinus pause	Does not meet the inclusion criteria.

Author(s)	Summary	Reason for Exclusion
	subsequent to taking nirmatrelvir/ritonavir.	
[218]	Report consensus on diagnosis and treatment of COVID-19 related cough and present nirmatrelvir/ritonavir as one treatment option.	Does not meet the inclusion criteria.
[219]	No abstract available on pubmed.	Abstract unavailable.
[220]	No abstract available on pubmed.	Abstract unavailable.
[221]	Explore the frequency of COVID-19's resistance to Nirmatrelvir/ritonavir and virological/clinical rebounds and underline the importance of these findings for immunocompromised patients.	Does not meet the inclusion criteria.
[222]	Discussion of the potential of theranostics regarding COVID-19 antivirals.	Does not meet the inclusion criteria.
[223]	Case report of a patient, who is being treated with clozapine and who, after receiving Paxlovid, developed neutropenia.	Does not meet the inclusion criteria.
[224]	Commentary on the differences, therapeutic potential, and best-practices of Paxlovid and Lagevrio to inform and support community pharmacy practices.	Does not meet the inclusion criteria.
[225]	Discovery of a uniquely Y-shaped, S-O-N-O-S-bridged post-translational cross-link that connects three residues C22, C44, and K61 at their side chains while analyzing M^{Pro} crystals.	Does not meet the inclusion criteria.
[226]	Case report of a patient who	Does not meet the inclusion

Author(s)	Summary	Reason for Exclusion
	experienced symptomatic bradycardia, likely as a result of a drug-drug interaction of ritonavir-boosted nirmatrelvir and verapamil.	criteria.
[227]	Essay on how to ease the process of obtaining Paxlovid for the treatment of COVID-19.	Does not meet the inclusion criteria.
[228]	Review of the treatment possibilities for COVID-19 in pediatric care. Come to the conclusion that molnupiravir cannot be used, while the other currently available antivirals may be administered at least to older children.	Does not meet the inclusion criteria.
[229]	No abstract available on pubmed.	Abstract unavailable.
[230]	No abstract available on pubmed.	Abstract unavailable.
[231]	Exploration of pediatric Nirmatrelvir/Ritonavir Prescribing Patterns, coming to the conclusion that Nirmatrelvir/Ritonavir is prescribed infrequently to children.	Does not meet the inclusion criteria.
[232]	No abstract available on pubmed.	Abstract unavailable.
[233]	No abstract available on pubmed.	Abstract unavailable.
[234]	No abstract available on pubmed.	Abstract unavailable.
[235]	Report a devised route to nirmatrelvir that reduces the overall sequence to a 1-pot process, relying on the green coupling reagent T3P.	Does not meet the inclusion criteria.
[236]	Presentation of some examples of new peptides and	Does not meet the inclusion criteria.

Author(s)	Summary	Reason for Exclusion
	peptidomimetic protease inhibitors in the field of SARS-CoV-2.	
[237]	Present the SARS-COV-2-within-host evolution of 15 patients during antiviral treatment, comparing their in vivo variability to drug-naive patients.	Does not meet the inclusion criteria.
[238]	Considers available literature and product information to assess potential drug-drug interactions between asthma medication and antiviral treatments for COVID-19.	Does not meet the inclusion criteria.
[239]	Case report of a patient who suffered from drug-drug interaction between tacrolimus and nirmatrelvir/ritonavir resulting in toxic levels of tacrolimus.	Does not meet the inclusion criteria.
[240]	Present examples of drugs co-administered with pharmacoenhancers (e.g. nirmatrelvir/ritonavir).	Does not meet the inclusion criteria.
[241]	Retrospective analysis of COVID-19 patients who were eligible for nirmatrelvir/ritonavir treatment during early 2022. Discussion of prescription rate, reasons for non-prescription as well as patient outcomes.	Does not meet the inclusion criteria.
[242]	Review which summarizes the basic features of proteaste of human immunodeficiency virus (HIV), hepatitis C virus (HCV), and SARS-CoV-2 and discusses the properties of their inhibitors in clinical use.	Does not meet the inclusion criteria.

Author(s)	Summary	Reason for Exclusion
[243]	No abstract available on pubmed.	Abstract unavailable.
[244]	Case report of an elderly patient who was being administered Paxlovid upon admittance to the hospital due to moderate COVID-19.	Does not meet inclusion criteria.
[245]	No abstract available on pubmed.	Abstract unavailable.
[246]	Acknowledge the importance of systematic, orderly and complete collection of health data of citizens and explore how the clinical research methodology process has changed in Italy due to COVID-19.	Does not meet the inclusion criteria.
[247]	Exploration of nirmatrelvir developmental toxicity using zebrafish as in vivo model to contribute to research on administration of nirmatrelvir during pregnancy.	Does not meet the inclusion criteria.
[248]	Explore the optimal docking between M^{pro} and antiviral drugs concluding that Paxlovid may work on new strains, including omicron as Omicrons M^{pro} mutation has no direct effect on the protein.	Does not meet the inclusion criteria.
[249]	Performance of in vitro and in vivo studies to explore the effect of nirmatrelvir/ritonavir on chondrocyte degeneration and senescence.	Does not meet the inclusion criteria.
[250]	Presentation of difficulties when administering nirmatrelvir/ritonavir to epilepsy patients.	Does not meet the inclusion criteria.
[251]	Assessment of potency of	Does not meet the inclusion

Author(s)	Summary	Reason for Exclusion
	molnupiravir and nirmatrelvir/ritonavir against SARS-Cov-2 in two animal models (Roborovski dwarf hamster model and the ferret model of upper respiratory tract disease and transmission) confirming similar therapeutic benefit of both drugs for older adults.	criteria.
[252]	Development of compartmental model, which describes SARS-CoV-2 spread after the reopening of a French Zero-COVID territory, which displays high vaccination hesitancy, low immunity, and high levels of comorbidities.	Does not meet the inclusion criteria.
[253]	Introduction of a new method to transparently report the living evidence surveillance process that occurs between published living systematic reviews (LSR) versions. Example used is a review on nirmatrelvir/ritonavir.	Does not meet the inclusion criteria.
[254]	No abstract available on pubmed.	Abstract unavailable.
[255]	Case report of a heart transplant recipients to increase awareness of nuances when treating COVID-19 for such patients.	Does not meet the inclusion criteria.
[256]	In silico study that suggests that sage is a rich source of potent anti-coronavirus flavonoids.	Does not meet the inclusion criteria.
[257]	Presentation of various COVID-19 treatments and therapies.	Does not meet the inclusion criteria.
[258]	Usage of *in silico* mutational scanning and inhibitor docking	Does not meet the inclusion criteria.

Author(s)	Summary	Reason for Exclusion
	of Mpro to identify potential resistance mutations.	
[259]	Case report of three children/adolescents who received Paxlovid to treat their COVID-19 infection. Paxlovid was effective and relatively safe in these three cases.	Does not meet the inclusion criteria.
[260]	Suggest that a direct application of 2-5 oligoadenylates, included in an inhalation spray, may be effective in treatment of severe COVID-19 infections.	Does not meet the inclusion criteria.
[261]	No abstract available on pubmed.	Abstract unavailable.
[98]	Investigation of the susceptibility of natural variants of SARS-CoV-2's main protease to monitor variance resistant to currently available vaccines and drugs.	Does not meet the inclusion criteria.
[262]	Uncover a metabolic pathway that simultaneously modulates pulmonary inflammation, tissue recovery and host metabolic health, which patients with an underlying metabolic disease suffering from COVID-19 are especially prone to.	Does not meet the inclusion criteria.
[263]	Analyze the effect of nirmatrelvir/ritonavir on the length of hospital stay, finding that Paxlovid reduces the viral clearance time and length of hospital stay for hospitalized COVID-19 patients.	Does not meet the inclusion criteria.
[264]	A review which explores the evidence of tested herocyclic	Does not meet the inclusion criteria.

Author(s)	Summary	Reason for Exclusion
	compounds acting on different targets against COVID-19 in order to help the development of novel COVID-19 drugs.	
[265]	Discuss and encourage the usage of convalescent plasma when treating immunocompromised COVID-19 patients.	Does not meet the inclusion criteria.
[266]	Review of currently approved drugs and most of the important drug candidates for COVID-19 related targets.	Does not meet inclusion criteria.
[267]	Description of a LC-MS/MS method for the simultaneous quantification of nirmatrelvir and ritonavir in human plasma of COVID-19 patients who are being treated with nirmatrelvir/ritonavir.	Does not meet the inclusion criteria.
[268]	No abstract available on pubmed.	Abstract unavailable.
[269]	Study of the solid-state behavior of the drugs ritonavir and lopinavir in raw materials and in milled compositions indicated that mixtures of ritonavir Forms I and II are found in different raw materials from the same manufacturer.	Does not meet the inclusion criteria.
[270]	Discussion of four patients with end-stage renal disease undergoing hemodialysis, who simultaneously received nirmatrelvir/ritonavir.	Does not meet the inclusion criteria.
[271]	Case report of a cardiac transplant patient who suffered from tracolimus toxicity induced by outpatient treatment for COVID-19 with	Does not meet the inclusion criteria.

Author(s)	Summary	Reason for Exclusion
	ritonavir-nirmatrelvir.	
[272]	Explore racial and ethnic disparities for nirmatrelvir/ritonavir prescriptions.	Does not meet the inclusion criteria.
[273]	Report pharmacists' experiences of managing/counseling/following up the usage of nirmatrelvir/ritonavir.	Does not meet the inclusion criteria.
[274]	Analyze nirmatrelvir/ritonavir and molnupiravir dispensing rates by zip code-level social vulnerability and highlight intervention strategies.	Does not meet the inclusion criteria.
[275]	Write an "A guide to" article to explain the development of antiviral drugs against SARS-CoV-2 and their potential in fighting COVID-19.	Does not meet the inclusion criteria.
[276]	Characterization of 14 naturally occurring M polymorphisms that are close to the binding site of these antivirals and testing of potency of nirmatrelvir and ensitrelvir against these polymorphisms.	Does not meet the inclusion criteria.
[277]	Explore the progress of circulating microRNAs in the regulation of severe inflammatory response, immune dysfunction, and thrombosis caused by SARS-CoV-2 infection.	Does not meet the inclusion criteria.
[278]	Presentation of a class of enzyme inhibitors, which could serve as a class of COVID-19 drug targets.	Does not meet the inclusion criteria.
[279]	Description of an in vitro pathway of SARS-CoV-2	Does not meet the inclusion criteria.

Author(s)	Summary	Reason for Exclusion
	development towards resistance against nirmatrelvir/ritonavir.	
[280]	Analyze recent mutations of Mpro coming to the conclusion that said increased mutational variability may encourage development of antiviral resistance.	Does not meet the inclusion criteria.
[281]	Present the current stage of development of covalent and noncovalent main protease (Mpro) inhibitors and the identification of nirmatrelvir-resistant mutants.	Does not meet the inclusion criteria.
[282]	Report of an optically active synthesis of nirmatelvir that avoids a critical epimerization step.	Does not meet the inclusion criteria.
[283]	Review on COVID-19 vaccines, focusing on their protective efficacies, as well as presentation of an overview of progress in developing potent drugs and monoclonal antibodies against COVID-19.	Does not meet the inclusion criteria.
[284]	Presentation of a COVID-19 oral antiviral assessment protocol developed by an independent community pharmacy and highlighting of the ability of community pharmacists to identify and address medication-related problems.	Does not meet the inclusion criteria.
[285]	Introduction of a nirmatrelvir/ritonavir physiologically-based pharmacokinetic (PBPK) model.	Does not meet the inclusion criteria.

Author(s)	Summary	Reason for Exclusion
	Does not meet inclusion criteria.	
[286]	Exploration of a rare natural mutation, H172Y and its potential ability to weaken nirmatrelvir's inhibitory activity.	Does not meet the inclusion criteria.
[287]	Updated version of the preprint above.	Does not meet the inclusion criteria.
[288]	Explore the imitation of the utility of pharmacists prescriptive authority for Paxlovid due the the restrictions included in the introduced FDA framework.	Does not meet the inclusion criteria.
[289]	Suggest the replacement of hydrogens with deuterium in nirmatrelvir to slow oxidation and potentially replace ritonavir, which alters the metabolism of other drugs	Does not meet the inclusion criteria.
[290]	Clinical trial of VV116, a potential alternative to nirmatrelvir/ritonavir for oral treatment of COVID-19.	Does not meet the inclusion criteria.
[291]	No abstract available on pubmed.	Abstract unavailable.
[292]	No abstract available on pubmed.	Abstract unavailable.
[293]	No abstract available on pubmed.	Abstract unavailable.
[294]	Usage of Density Functional Theory to examine electronic and thermodynamic properties of nine COVID-19 drugs, coming to the conclusion that the electrophilic indexes are important to understand their efficiencies.	Does not meet the inclusion criteria.
[295]	Review of current knowledge	Does not meet the inclusion

Author(s)	Summary	Reason for Exclusion
	of pathogenesis and neurological disorders related to COVID-19 to determine potential of using Panax ginseng to treat neurological disorders.	criteria.
[296]	Review on the currently employed COVID-19 management approaches and discussion of available treatment and prevention strategies.	Does not meet the inclusion criteria.
[297]	Description of the uptake and delivery of intravenous monoclonal antibodies, nirmatrelvir-ritonavir, and molnupiravir in the Torres and Cape region.	Does not meet the inclusion criteria.
[298]	Review of the current challenges of available COVID-19 treatments and drug development strategies for future therapy.	Does not meet the inclusion criteria.
[299]	Clinical trial finding that nirmatrelvir/ritonavir can significantly reduce the viral shedding time in elderly Chinese patients.	Does not meet the inclusion criteria.
[300]	No abstract available on pubmed.	Abstract unavailable.
[301]	No abstract available on pubmed.	Abstract unavailable.
[302]	Presentation of a patent of novel proline derivatives for the treatment of COVID-19.	Does not meet the inclusion criteria.
[303]	Discussion of access barriers to Paxlovid in low- and middle-income countries.	Does not meet the inclusion criteria.
[304]	No abstract available on pubmed.	Abstract unavailable.

Author(s)	Summary	Reason for Exclusion
[305]	Editorial with the aim of providing an update on current knowledge on COVID-19 rebound and how this may relate to currently available drugs.	Does not meet the inclusion criteria.
[306]	No abstract available on pubmed.	Abstract unavailable.
[307]	No abstract available on pubmed.	Abstract unavailable.
[308]	No abstract available on pubmed.	Abstract unavailable.
[309]	Report of 14 cases of COVID-19 patients, who are kidney transplant recipients coming to the conclusion that nirmatrelvir/ritonavir can be used in these patients, but should be closely monitored.	Does not meet the inclusion criteria.
[310]	Report of a cohort of 483 high-risk COVID-19 patients, who were being treated nirmatrelvir-ritonavir and of whom 2 required hospitalization, while 4 experienced rebound symptoms	Does not meet the inclusion criteria.
[311]	No abstract available on pubmed.	Abstract unavailable.
[312]	No abstract available on pubmed.	Abstract unavailable.
[313]	Examine the effect of nirmatrelvir/ritonavir and Chinese herb medicine lianhuaqingwen on PCR conversion time finding that early and aggressive administration of Paxlovid can significantly reduce the PCR conversion time.	Does not meet the inclusion criteria.

Author(s)	Summary	Reason for Exclusion
[314]	Investigation of the MEK1/2 inhibitor ATR-002's ability to potentiate the effect of direct-acting antivirals (DAA) against SARS-CoV-2 on viral replication.	Does not meet the inclusion criteria.
[315]	Identification of 100 naturally occurring M^{pro} mutations, located at the nirmatrelvir binding site, which might consequently inhibit Paxlovid's efficacy.	Does not meet the inclusion criteria.
[316]	Retrospective cohort study comparing the rate of progression to severe disease after receiving bebtelovimab or nirmatrelvir-ritonavir, coming to the conclusion that betelovimab's efficacy was not significantly different from nirmatrelvir/ritonavir's.	Does not meet the inclusion criteria.
[317]	Explore how nanotechnology provides potential alternative solutions to combat COVID-19.	Does not meet the inclusion criteria.
[318]	To improve currently available COVID-19 drugs, the authors suggest a combination of host- and virus-targeting antiviral drugs as it has the potential to synergistically suppress SARS-CoV-2 infection in Calu-3 lung epithelial cells.	Does not meet the inclusion criteria.
[319]	Review of the advances and challenges related to using nirmatrelvir to treat COVID-19 in order to provide insight to both drug developers and physicians treating COVID-19 patients.	Does not meet the inclusion criteria.
[320]	Review on the progress in	Does not meet the inclusion

Author(s)	Summary	Reason for Exclusion
	development of peptide drugs for use in the treatment of COVID-19.	criteria.
[321]	Evaluation of the trimeric derivative TF27 for its anti-SARS-CoV-2 potential coming to the conclusion that trioxane compounds should be considered for pharmacologic development towards future COVID-19 medication.	Does not meet the inclusion criteria.
[322]	Exploration of plant-derived antiviral small molecules to halt the viral entrance and in consequence serve as a component of future COVID-19 drugs.	Does not meet the inclusion criteria.
[323]	Description of the protocol for the Pediatric Observational Cohort Study of the NIH's **RE** searching **COV** ID to **E** nhance **R** ecovery (RECOVER) Initiative, focusing on two of five cohorts.	Does not meet the inclusion criteria.
[324]	Argue that nirmatrelvir/ritonavir and molnupiravir therapy is frequently underutilized. To overcome this underutilization, they describe the implementation of a pharmacy consult service for oral COVID-19 drugs within the ambulatory care setting.	Does not meet the inclusion criteria.
[325]	Structurally analyze the coronavirus main protease, focus on frequently occurring atoms and residues to find a generalized strategy for inhibitor design given a large	Does not meet the inclusion criteria.

Author(s)	Summary	Reason for Exclusion
	amount of protein complex from SARS-Cov.	
[326]	Long-term study of virological outcomes of immunodeficient individuals to uncover their (lack of) ability to sustain viral clearance.	Does not meet the inclusion criteria.
[327]	Discuss the possibility to reduce the ritonavir dosage to lower unwanted side effects and introduce another potent CYP3A inhibitor, cobicistat.	Does not meet the inclusion criteria.
[328]	Query the French national pharmacovigilance database to better understand nirmatrelvir/ritonavir's drug safety profile and potentially associated drug-drug interactions.	Does not meet the inclusion criteria.
[329]	Investigation of both GC376, a transition-state analog inhibitor of the main protease of feline infectious peritonitis coronavirus, and Nirmatrelvir to find that both are capable of strongly binding SARS-CoV-2's M^{pro}.	Does not meet the inclusion criteria.
[330]	Pursue an AI-driven approach followed by in vitro validation, to identify five fragment like M^{Pro} inhibitors.	Does not meet the inclusion criteria.
[331]	Introduce latest version of COVID-19Base, a database summarizing the biomedical entities of COVID-19.	Does not meet the inclusion criteria.
[332]	Review summarizing the current developments in plant-derived drugs to combat COVID-19.	Does not meet the inclusion criteria.
[333]	Argue for a global Intellectual	Does not meet the inclusion

Author(s)	Summary	Reason for Exclusion
	Property waiver to meet the ongoing inequities of COVID-19, and to ensure equitable pandemic preparedness.	criteria.
[334]	Analysis of 213 critical, aged 50 years or younger, adult COVID-19 patients in South Korea to determine clinical characteristics and risk factors for mortality.	Does not meet the inclusion criteria.
[335]	Usage of virtual screening, molecular docking, and molecular dynamics simulations to identify potent phytocompounds as inhibitors of SARS-CoV-2 targets.	Does not meet the inclusion criteria.
[336]	Retrospectively analyze routine clinical data from outpatients with COVID-19 at high risk of severe outcomes to ascertain patient eligibility status and describe coverage of antiviral drugs and neutralising monoclonal antibodies (nMAB) as treatment for COVID-19 in community settings.	Does not meet the inclusion criteria.
[337]	Identification of prevalence of $3CL^{Pro}$ inhibitor mutations that are associated with reduced susceptibility to nirmatrelvir/ritonavir.	Does not meet the inclusion criteria.
[338]	Descriptive study baked on the French Pharmacovigilance Database to determine adverse drug reactions in COVID-19 patients.	Does not meet the inclusion criteria.
[339]	Review on the recent literature on drugs which were at the time under advanced clinical evaluation for COVID-19	Does not meet the inclusion criteria.

Author(s)	Summary	Reason for Exclusion
	treatment. They include antiviral agents as well as host-directed therapies.	
[340]	Usage of a network-based bioinformatics methodology to investigate the potential side effects of known Food and Drug Administration (FDA)-approved antihypertensive drugs, Sartans as well as application of the same methodology to nirmatrelvir/ritonavir.	Does not meet the inclusion criteria.
[341]	Report the inhibitory potentials of epigenetic-targeting drugs against SARS-CoV-2's M^{pro}.	Does not meet the inclusion criteria.
[342]	Application of in vitro screen using the 2560 compounds from the Microsource Spectrum library to identify two additional small-molecule hits that can serve as PL^{pro} and M^{pro} inhibitors.	Does not meet the inclusion criteria.
[343]	Application of population pharmacokinetic modeling with clinical study data was employed to explore the pharmacokinetic profile of nirmatrelvir in older adult Chinese patients with COVID-19 infection	Does not meet the inclusion criteria.
[344]	Identification of the top 100 drugs most commonly prescribed to US patients who have a high risk of progressing to severe COVID-19. Each of these 100 drugs on the one hand and nirmatrelvir/ritonavir on the other hand were then examined for their drug-drug	Does not meet the inclusion criteria.

Author(s)	Summary	Reason for Exclusion
	interaction risk.	
[345]	Discussion of aspects of immune response in order to understand B and T cell lymphocyte regulatory mechanisms and antibody effectiveness and senescence plus presentation of COVID-19 therapies for non-hospitalized patients including limitations for identification, collection and distribution to infected patients.	Does not meet the inclusion criteria.
[346]	Screening of prognostic signature of lung adenocarcinoma/COVID-19 patients to identify the potential therapeutic targets and molecular pathways of nirmatrelvir/ritonavir.	Does not meet the inclusion criteria.
[347]	Discover subtle structural differences of nucleotide analogs that might lead to more potent SARS-CoV-2 inhibitors than the ones used in currently available COVID-19 drugs.	Does not meet the inclusion criteria.
[348]	Summary of literature, which was available at the time of publication, on nirmatrelvir/ritonavir, focusing on dosage, drug-drug interactions, toleration as well as how nirmatelvir/ritonavir combats COVID-19 infections. Only efficacy study included is the clinical trial by [9].	Does not meet the inclusion criteria.
[349]	Review summarizing the clinical outcomes that have been obtained from the clinical trials published on antivirals,	Does not meet the inclusion criteria.

Author(s)	Summary	Reason for Exclusion
	immunomodulators, and other medications, such as antibiotics, stem cells, and plasma therapy in the treatment of COVID-19.	
[350]	Review on efficacy and safety of nirmatrelvir/ritonavir in, includes studies up until July 11, 2022.	Does not meet the inclusion criteria.
[351]	Review on nirmatrelvir/ritonavir. Explains how nirmatrelvir can inhibit SARS-CoV-2's M^{Pro} , how ritonavir increases plasma concentration of nirmatrelvir, introduces the associated clinical studies and discusses possible safety concerns, the need for more studies who explore drug-drug interactions. Different focus than our work.	Does not meet the inclusion criteria.
[352]	Meta-analysis of relevant studies up to March, 2023 to estimate nirmatelvir/ritonavir's efficacy compared with other antiviral drugs. Hence, their focus differs from ours.	Does not meet the inclusion criteria.
[353]	Review on efficacy and safety of nirmatrelvir/ritonavir. Control group consisted of patients who did not receive any COVID-19 medication. Included articles up to January 1, 2023. Hence, the studies we present from 2023 are not taken into account here.	Does not meet the inclusion criteria.
[354]	Review on efficacy and safety of nirmatrelvir/ritonavir. Included articles up to November 10, 2022. Hence, the	Does not meet the inclusion criteria.

Author(s)	Summary	Reason for Exclusion
	studies we present from 2023 are not taken into account here.	
[355]	Meta-analysis on efficacy and safety of nirmatrelvir/ritonavir. Included articles up to February 15, 2023. The studies we present from 2023 are not taken into account.	Does not meet the inclusion criteria
[356]	Review which presents currently available findings in the development of small molecules for COVID-19 treatment.	Does not meet the inclusion criteria.
[357]	Review on current knowledge of Paxlovid to explore how it may be used to treat future COVID-19 patients.	Does not meet the inclusion criteria.
[3]	Examine disparities in Paxlovid treatment, finding lower rates among Black and Hispanic or Latino patients and within socially vulnerable communities.<br>Does not meet inclusion criteria.	Does not meet the inclusion criteria.
[358]	Review which summarizes Paxlovid's development journeys, undertaken clinical studies, regulatory approvals as well as current clinical trials and safety measures to end up with a discussion of drug-drug interactions and rebound effects.	Does not meet the inclusion criteria.
[359]	No abstract available on pubmed.	Does not meet the inclusion criteria.
[360]	Report of a phase 2/phase study of nirmatrelvir/ritonavir on patients with standard risk for developing severe COVID-19.	Does not meet the inclusion criteria.

Author(s)	Summary	Reason for Exclusion
	Here, the primary endpoint was self-reported sustained alleviation of all symptoms after 4 consecutive days. This endpoint was not met.	
[361]	Study on the efficacy of Paxlovid in elderly patients considering length of hospital stay, viral shedding time and rebound as outcomes.	Does not meet the inclusion criteria.
[362]	Review and discussion of efficacy and safety of nirmatrelvir/ritonavir by presenting the literature which was available at the time of publication. Does not include novel publications from 2023.	Does not meet the inclusion criteria.

References

1. Alsaeed A, Alkhalaf A, Alomran A, Alsfyani W, Alhaddad F, Alhaddad MJ (2023) Paxlovid for Treating COVID-19 Patients: A Case-Control Study From Two Hospitals in the Eastern Province of Saudi Arabia. Cureus 15:e39234
2. Arbel R, Wolff Sagy Y, Hoshen M, et al (2022) Nirmatrelvir Use and Severe Covid-19 Outcomes during the Omicron Surge. N Engl J Med 387:790–798
3. Bhatia A, Preiss AJ, Xiao X, et al (2023) Effect of Nirmatrelvir/Ritonavir (Paxlovid) on Hospitalization among Adults with COVID-19: an EHR-based Target Trial Emulation from N3C. medRxiv. https://doi.org/10.1101/2023.05.03.23289084
4. Cai H, Yan J, Liu S, et al (2023) Paxlovid for hospitalized COVID-19 patients with chronic kidney disease. Antiviral Res 216:105659
5. Cegolon L, Pol R, Simonetti O, Larese Filon F, Luzzati R (2023) Molnupiravir, Nirmatrelvir/Ritonavir, or Sotrovimab for High-Risk COVID-19 Patients Infected by the Omicron Variant: Hospitalization, Mortality, and Time until Negative Swab Test in Real Life. Pharmaceuticals . https://doi.org/10.3390/ph16050721
6. Dryden-Peterson S, Kim A, Kim AY, et al (2023) Nirmatrelvir Plus Ritonavir for Early COVID-19 in a Large U.S. Health System : A Population-Based Cohort Study. Ann Intern Med 176:77–84
7. Faust JS, Kumar A, Shah J, Khadke S, Dani SS, Ganatra S, Sax PE (2023) Oral Nirmatrelvir and Ritonavir for Covid-19 in Vaccinated, Non-Hospitalized Adults, Ages 18-50 Years. Clin Infect Dis. https://doi.org/10.1093/cid/ciad400
8. Ganatra S, Dani SS, Ahmad J, Kumar A, Shah J, Abraham GM, McQuillen DP, Wachter RM, Sax PE (2022) Oral Nirmatrelvir and Ritonavir in Non-hospitalized Vaccinated Patients with Covid-19. Clin Infect Dis. https://doi.org/10.1093/cid/ciac673
9. Hammond J, Leister-Tebbe H, Gardner A, et al (2022) Oral Nirmatrelvir for High-Risk, Nonhospitalized Adults with Covid-19. N Engl J Med 386:1397–1408
10. Hansen K, Makkar SR, Sahner D, Fessel J, Hotaling N, Sidky H (2023) Paxlovid (nirmatrelvir/ritonavir) effectiveness against hospitalization and death in N3C: A target trial emulation study. medRxiv. https://doi.org/10.1101/2023.05.26.23290602
11. Kim JM, Yoo M-G, Bae SJ, Kim J, Lee H (2023) Effectiveness of Paxlovid, an Oral Antiviral Drug, Against the Omicron BA.5 Variant in Korea: Severe Progression and Death Between July and November 2022. J Korean Med Sci 38:e211
12. Liu J, Pan X, Zhang S, et al (2023) Efficacy and safety of Paxlovid in severe adult patients with SARS-Cov-2 infection: a multicenter randomized controlled study. Lancet Reg Health West Pac 33:100694
13. Najjar-Debbiny R, Gronich N, Weber G, Khoury J, Amar M, Stein N, Goldstein LH, Saliba W (2022) Effectiveness of Paxlovid in Reducing Severe COVID-19 and Mortality in High Risk Patients. Clin Infect Dis. https://doi.org/10.1093/cid/ciac443
14. Park H, Park YJ, Lee HY, Yu M, Song Y-J, Lee SE, Lee J-J, Lee E-S, Kim Y (2022) The effectiveness of Paxlovid treatment in long-term care facilities in South Korea during the outbreak of the Omicron variant of SARS-CoV-2. Osong Public Health Res Perspect 13:443–447

15. Shah MM, Joyce B, Plumb ID, et al (2023) Paxlovid associated with decreased hospitalization rate among adults with COVID-19 - United States, April-September 2022. Am J Transplant 23:150–155
16. Shao J, Fan R, Guo C, Huang X, Guo R, Zhang F, Hu J, Huang G, Cao L (2023) Composite Interventions on Outcomes of Severely and Critically Ill Patients with COVID-19 in Shanghai, China. Microorganisms. https://doi.org/10.3390/microorganisms11071859
17. Wong CKH, Au ICH, Lau KTK, Lau EHY, Cowling BJ, Leung GM (2022) Real-world effectiveness of early molnupiravir and nirmatrelvir/ritonavir among hospitalized, non-oxygen-dependent COVID-19 patients on admission during Hong Kong's Omicron BA.2 wave: an observational study. https://doi.org/10.1101/2022.05.19.22275291
18. Epling BP, Rocco JM, Boswell KL, et al (2022) COVID-19 redux: clinical, virologic, and immunologic evaluation of clinical rebound after nirmatrelvir/ritonavir. medRxiv. https://doi.org/10.1101/2022.06.16.22276392
19. Extance A (2022) Covid-19: What is the evidence for the antiviral Paxlovid? BMJ 377:o1037
20. Fishbane S, Hirsch JS, Nair V (2022) Special Considerations for Paxlovid Treatment Among Transplant Recipients With SARS-CoV-2 Infection. Am J Kidney Dis 79:480–482
21. Gentile I, Scotto R, Moriello NS, et al (2022) Nirmatrelvir/ritonavir and molnuipiravir in the treatment of mild/moderate COVID-19: results of a real-life study. bioRxiv. https://doi.org/10.1101/2022.08.23.22278585
22. Wang L, Volkow ND, Davis PB, Berger NA, Kaelber DC, Xu R (2022) COVID-19 rebound after Paxlovid treatment during Omicron BA.5 vs BA.2.12.1 subvariant predominance period. https://doi.org/10.1101/2022.08.04.22278450
23. Marzolini C, Kuritzkes DR, Marra F, et al (2022) Recommendations for the Management of Drug-Drug Interactions Between the COVID-19 Antiviral Nirmatrelvir/Ritonavir (Paxlovid) and Comedications. Clin Pharmacol Ther. https://doi.org/10.1002/cpt.2646
24. Prikis M, Cameron A (2022) Paxlovid (Nirmatelvir/Ritonavir) and Tacrolimus Drug-Drug Interaction in a Kidney Transplant Patient with SARS-2-CoV infection: A Case Report. Transplant Proc. https://doi.org/10.1016/j.transproceed.2022.04.015
25. Rubin R (2022) From Positive to Negative to Positive Again-The Mystery of Why COVID-19 Rebounds in Some Patients Who Take Paxlovid. JAMA 327:2380–2382
26. Vangeel L, Chiu W, De Jonghe S, Maes P, Slechten B, Raymenants J, André E, Leyssen P, Neyts J, Jochmans D (2022) Remdesivir, Molnupiravir and Nirmatrelvir remain active against SARS-CoV-2 Omicron and other variants of concern. Antiviral Res 198:105252
27. Wen W, Chen C, Tang J, et al (2022) Efficacy and safety of three new oral antiviral treatment (molnupiravir, fluvoxamine and Paxlovid) for COVID-19：a meta-analysis. Ann Med 54:516–523
28. Abdelrahim M, Esmail A, Al Saadi N, Zsigmond E, Al Najjar E, Bugazia D, Al-Rawi H, Alsaadi A, Kaseb AO (2022) Thymoquinone's Antiviral Effects: It is Time to be Proven in the Covid-19 Pandemic Era and its Omicron Variant Surge. Front Pharmacol 13:848676
29. Alshanqeeti S, Bhargava A (2022) COVID-19 Rebound After Paxlovid Treatment: A Case Series and Review of Literature. Cureus 14:e26239
30. Alvarado YJ, Olivarez Y, Lossada C, et al (2022) Interaction of the new inhibitor paxlovid (PF-07321332) and ivermectin with the monomer of the main protease SARS-CoV-2: A volumetric study based on molecular dynamics, elastic networks, classical thermodynamics and SPT. Comput Biol Chem 99:107692
31. Ashour NA, Abo Elmaaty A, Sarhan AA, Elkaeed EB, Moussa AM, Erfan IA, Al-Karmalawy AA (2022) A Systematic Review of the Global Intervention for SARS-CoV-2 Combating: From Drugs Repurposing to Molnupiravir Approval. Drug Des Devel Ther 16:685–715
32. Atluri K, Aimlin I, Arora S (2022) Current Effective Therapeutics in Management of COVID-19. J Clin Med Res. https://doi.org/10.3390/jcm11133838
33. Azanza JR, Mensa J, González Del Castillo J, Linares Rufo M, Molero JM, Madero Valle N, Barberán J (2022) Interactions listed in the Paxlovid fact sheet, classified according to risks, pharmacological groups, and consequences. Rev Esp Quimioter 35:357–361
34. Bartha FA, Juhász N, Marzban S, Han R, Röst G (2022) In Silico Evaluation of Paxlovid's Pharmacometrics for SARS-CoV-2: A Multiscale Approach. Viruses. https://doi.org/10.3390/v14051103
35. Ben Hlima H, Farhat A, Akermi S, Khemakhem B, Ben Halima Y, Michaud P, Fendri I, Abdelkafi S (2022) In silico evidence of antiviral activity against SARS-CoV-2 main protease of oligosaccharides from Porphyridium sp. Sci Total Environ 836:155580
36. Berar Yanay N, Bogner I, Saker K, Tannous E (2022) Paxlovid-Tacrolimus Drug-Drug Interaction in a 23-Year-Old Female Kidney Transplant Patient with COVID-19. Clin Drug Investig 42:693–695
37. Birabaharan M, Martin TCS (2022) Acute pulmonary emboli following rebound phenomenon after Nirmatrelvir/Ritonavir treatment for COVID-19. Am J Emerg Med. https://doi.org/10.1016/j.ajem.2022.08.012
38. Borio LL, Bright RA, Emanuel EJ (2022) A National Strategy for COVID-19 Medical Countermeasures: Vaccines and Therapeutics. JAMA 327:215–216
39. Brooks JK, Song JH, Sultan AS (2022) Paxlovid-associated dysgeusia. Oral Dis. https://doi.org/10.1111/odi.14312
40. Burki T (2022) The future of Paxlovid for COVID-19. Lancet Respir Med 10:e68
41. Buxeraud J, Faure S, Fougere É (2022) [Nirmatrelvir/ritonavir (Paxlovid®), a treatment for Covid-19]. Actual Pharm 61:10–12
42. Callaway E (2022) COVID rebound is surprisingly common - even without Paxlovid. Nature. https://doi.org/10.1038/d41586-022-02121-z

43. Catlin NR, Bowman CJ, Campion SN, Cheung JR, Nowland WS, Sathish JG, Stethem CM, Updyke L, Cappon GD (2022) Reproductive and developmental safety of nirmatrelvir (PF-07321332), an oral SARS-CoV-2 Mpro inhibitor in animal models. Reprod Toxicol 108:56–61
44. Cerón-Carrasco JP (2022) When Virtual Screening Yields Inactive Drugs: Dealing with False Theoretical Friends. ChemMedChem 17:e202200278
45. Chen H-Y, Chang R, Wei JCC (2022) Epidemiological study on the effectiveness of Paxlovid. Clin Infect Dis. https://doi.org/10.1093/cid/ciac664
46. Choi JH, Lee YH, Kwon TW, Ko S-G, Nah S-Y, Cho I-H (2022) Can Panax ginseng help control cytokine storm in COVID-19? J Ginseng Res 46:337–347
47. Collaborative TO, The OpenSAFELY Collaborative, Green A, et al (2022) Trends, variation and clinical characteristics of recipients of antivirals and neutralising monoclonal antibodies for non-hospitalised COVID-19: a descriptive cohort study of 23.4 million people in OpenSAFELY. https://doi.org/10.1101/2022.03.07.22272026
48. Coulson JM, Adams A, Gray LA, Evans A (2022) COVID-19 "Rebound" associated with nirmatrelvir/ritonavir pre-hospital therapy. J Infect 85:436–480
49. Couzin-Frankel J (2021) Antiviral pills could change pandemic's course. Science 374:799–800
50. Dai EY, Lee KA, Nathanson AB, et al (2022) Viral Kinetics of Severe Acute Respiratory Syndrome Coronavirus 2 (SARS-CoV-2) Omicron Infection in mRNA-Vaccinated Individuals Treated and Not Treated with Nirmatrelvir-Ritonavir. medRxiv. https://doi.org/10.1101/2022.08.04.22278378
51. de Oliveira V, Ibrahim M, Sun X, Hilgenfeld R, Shen J (2022) H172Y mutation perturbs the S1 pocket and nirmatrelvir binding of SARS-CoV-2 main protease through a nonnative hydrogen bond. Res Sq. https://doi.org/10.21203/rs.3.rs-1915291/v1
52. Deo R, Choudhary MC, Moser C, et al (2022) Viral and Symptom Rebound in Untreated COVID-19 Infection. medRxiv. https://doi.org/10.1101/2022.08.01.22278278
53. Drożdżal S, Rosik J, Lechowicz K, Machaj F, Szostak B, Przybyciński J, Lorzadeh S, Kotfis K, Ghavami S, Łos MJ (2021) An update on drugs with therapeutic potential for SARS-CoV-2 (COVID-19) treatment. Drug Resist Updat 59:100794
54. Rubin EJ, Baden LR, Morrissey S (2022) Audio Interview: A New Antiviral against Covid-19. N Engl J Med 386:e25
55. Eng H, Dantonio AL, Kadar EP, et al (2022) Disposition of Nirmatrelvir, an Orally Bioavailable Inhibitor of SARS-CoV-2 3C-Like Protease, across Animals and Humans. Drug Metab Dispos 50:576–590
56. Feingold KR (2022) Lipid and Lipoprotein Levels in Patients with COVID-19 Infections. Endotext
57. Feng Y, Liu Y, Liu L, Liu Y, Jiang Y, Hou Y, Zhou Y, Song R, Chen X, Wang X (2022) Real-world effectiveness of Yindan Jiedu granules-based treatment on patients infected with the SARS-CoV-2 Omicron variants BA.2 combined with high-risk factors: A cohort study. Front Pharmacol 13:978979
58. Fernando K, Menon S, Jansen K, Naik P, Nucci G, Roberts J, Wu SS, Dolsten M (2022) Achieving end-to-end success in the clinic: Pfizer's learnings on R&D productivity. Drug Discov Today 27:697–704
59. Ferrara F, Zovi A, Trama U, Vitiello A (2022) Nirmatrelvir-remdesivir association for non-hospitalized adults with COVID-19, point of view. Inflammopharmacology 30:1927–1931
60. Gandhi RT, Malani PN, Del Rio C (2022) COVID-19 Therapeutics for Nonhospitalized Patients. JAMA 327:617–618
61. García-Lledó A, Gómez-Pavón J, González Del Castillo J, et al (2022) Pharmacological treatment of COVID-19: an opinion paper. Rev Esp Quimioter 35:115–130
62. Gold JAW, Kelleher J, Magid J, et al (2022) Dispensing of Oral Antiviral Drugs for Treatment of COVID-19 by Zip Code-Level Social Vulnerability - United States, December 23, 2021-May 21, 2022. MMWR Morb Mortal Wkly Rep 71:825–829
63. Greasley SE, Noell S, Plotnikova O, et al (2022) Structural basis for the in vitro efficacy of nirmatrelvir against SARS-CoV-2 variants. J Biol Chem 298:101972
64. Halford B (2022) The Path to Paxlovid. ACS Cent Sci 8:405–407
65. Ho WS, Zhang R, Tan YL, Chai CLL (2022) COVID-19 and the promise of small molecule therapeutics: Are there lessons to be learnt? Pharmacol Res 179:106201
66. Hong E, Almond LM, Chung PS, Rao AP, Beringer PM (2022) Physiologically-Based Pharmacokinetic-Led Guidance for Patients With Cystic Fibrosis Taking Elexacaftor-Tezacaftor-Ivacaftor With Nirmatrelvir-Ritonavir for the Treatment of COVID-19. Clin Pharmacol Ther 111:1324–1333
67. Huang J, Yin D, Qin X, Yu M, Jiang B, Chen J, Cao Q, Tang Z (2022) Case report: Application of nirmatrelvir/ritonavir to treat COVID-19 in a severe aplastic anemia child after allogeneic hematopoietic stem cell transplantation. Front Pediatr 10:935118
68. Hung Y-P, Lee J-C, Chiu C-W, Lee C-C, Tsai P-J, Hsu I-L, Ko W-C (2022) Oral Nirmatrelvir/Ritonavir Therapy for COVID-19: The Dawn in the Dark? Antibiotics (Basel). https://doi.org/10.3390/antibiotics11020220
69. Islam T, Hasan M, Rahman MS, Islam MR (2022) Comparative evaluation of authorized drugs for treating Covid-19 patients. Health Sci Rep 5:e671
70. Javaux C, Ader F (2022) [Prise en charge médicale du Covid-19, hors réanimation]. Rev Prat 72:505–510
71. Joyce RP, Hu VW, Wang J (2022) The history, mechanism, and perspectives of nirmatrelvir (PF-07321332): an orally bioavailable main protease inhibitor used in combination with ritonavir to reduce COVID-19-related hospitalizations. Med Chem Res 31:1637–1646
72. Katella K (2022) 13 Things To Know About Paxlovid, the Latest COVID-19 Pill. In: yalemedicine.org. https://www.yalemedicine.org/news/13-things-to-know-paxlovid-covid-19. Accessed 4 Aug 2022

73. Kozlov M (2022) Why scientists are racing to develop more COVID antivirals. Nature 601:496
74. Kuehn BM (2022) Rehospitalization, Emergency Visits After Paxlovid Treatment Are Rare. JAMA 328:323
75. Wang L, Berger NA, Davis PB, Kaelber DC, Volkow ND, Xu R (2022) COVID-19 rebound after Paxlovid and Molnupiravir during January-June 2022. medRxiv. https://doi.org/10.1101/2022.06.21.22276724
76. Lamb YN (2022) Nirmatrelvir Plus Ritonavir: First Approval. Drugs 82:585–591
77. Ledford H, Maxmen A (2022) African clinical trial denied access to key COVID drug Paxlovid. Nature 604:412–413
78. Ledford H (2022) Hundreds of COVID trials could provide a deluge of new drugs. Nature 603:25–27
79. Lee JT, Yang Q, Gribenko A, Perrin BS Jr, Zhu Y, Cardin R, Liberator PA, Anderson AS, Hao L (2022) Genetic Surveillance of SARS-CoV-2 Mpro Reveals High Sequence and Structural Conservation Prior to the Introduction of Protease Inhibitor Paxlovid. MBio 13:e0086922
80. Lemaitre F, Budde K, Van Gelder T, et al (2022) Therapeutic drug monitoring and dosage adjustments of immunosuppressive drugs when combined with nirmatrelvir/ritonavir in patients with COVID-19. Ther Drug Monit. https://doi.org/10.1097/FTD.0000000000001014
81. Lemaitre F, Grégoire M, Monchaud C, et al (2022) Management of drug-drug interactions with nirmatrelvir/ritonavir in patients treated for Covid-19: Guidelines from the French Society of Pharmacology and Therapeutics (SFPT). Therapie. https://doi.org/10.1016/j.therap.2022.03.005
82. Li P, Wang Y, Lavrijsen M, Lamers MM, de Vries AC, Rottier RJ, Bruno MJ, Peppelenbosch MP, Haagmans BL, Pan Q (2022) SARS-CoV-2 Omicron variant is highly sensitive to molnupiravir, nirmatrelvir, and the combination. Cell Res 32:322–324
83. Lieber CM, Plemper RK (2022) 4′-Fluorouridine Is a Broad-Spectrum Orally Available First-Line Antiviral That May Improve Pandemic Preparedness. DNA Cell Biol 41:699–704
84. Liu C, Yan W, Shi J, Wang S, Peng A, Chen Y, Huang K (2022) Biological Actions, Implications, and Cautions of Statins Therapy in COVID-19. Front Nutr 9:927092
85. Liu C, Zhu M, Cao L, Boucetta H, Song M, Hang T, Lu Y (2022) Simultaneous determination of nirmatrelvir and ritonavir in human plasma using LC-MS/MS and its pharmacokinetic application in healthy Chinese volunteers. Biomed Chromatogr e5456
86. Logue J, Chakraborty AR, Johnson R, et al (2022) PIKfyve-specific inhibitors restrict replication of multiple coronaviruses in vitro but not in a murine model of COVID-19. Commun Biol 5:808
87. Lu G, Zhang Y, Zhang H, et al (2022) Geriatric risk and protective factors for serious COVID-19 outcomes among older adults in Shanghai Omicron wave. Emerg Microbes Infect 11:2045–2054
88. Mahaboob Ali AA, Bugarcic A, Naumovski N, Ghildyal R (2022) Ayurvedic formulations: Potential COVID-19 therapeutics? Phytomed Plus 2:100286
89. Mahase E (2021) Covid-19: Pfizer's paxlovid is 89% effective in patients at risk of serious illness, company reports. BMJ 375:n2713
90. Malden DE, Hong V, Lewin BJ, Ackerson BK, Lipsitch M, Lewnard JA, Tartof SY (2022) Hospitalization and Emergency Department Encounters for COVID-19 After Paxlovid Treatment - California, December 2021-May 2022. MMWR Morb Mortal Wkly Rep 71:830–833
91. Manus J-M (2022) Pfizer, résultats très prometteurs de l'antiviral oral paxlovid. Rev Francoph Lab 2022:5
92. Marzi M, Vakil MK, Bahmanyar M, Zarenezhad E (2022) Paxlovid: Mechanism of Action, Synthesis, and In Silico Study. Biomed Res Int 2022:7341493
93. McDonald EG, Lee TC (2022) Nirmatrelvir-ritonavir for COVID-19. CMAJ 194:E218
94. McDonald EG, Lee TC (2022) Association nirmatrelvir/ritonavir contre la COVID-19. CMAJ 194:E365–E366
95. McMillan NAJ, Morris KV, Idris A (2022) RNAi to treat SARS-CoV-2-variant proofing the next generation of therapies. EMBO Mol Med 14:e15811
96. Chang Y-C, Yang C-F, Chen Y-F, et al (2022) A siRNA targets and inhibits a broad range of SARS-CoV-2 infections including Delta variant. EMBO Mol Med 14:e15298
97. Mikus G, Foerster KI, Terstegen T, Vogt C, Said A, Schulz M, Haefeli WE (2022) Oral Drugs Against COVID-19. Dtsch Arztebl Int 119:263–269
98. Moghadasi SA, Heilmann E, Moraes SN, Kearns FL, von Laer D, Amaro RE, Harris RS (2022) Transmissible SARS-CoV-2 variants with resistance to clinical protease inhibitors. bioRxiv. https://doi.org/10.1101/2022.08.07.503099
99. Mohapatra RK, Tiwari R, Sarangi AK, Islam MR, Chakraborty C, Dhama K (2022) Omicron (B.1.1.529) variant of SARS-CoV-2: Concerns, challenges, and recent updates. J Med Virol 94:2336–2342
100. Mótyán JA, Mahdi M, Hoffka G, Tőzsér J (2022) Potential Resistance of SARS-CoV-2 Main Protease (Mpro) against Protease Inhibitors: Lessons Learned from HIV-1 Protease. Int J Mol Sci. https://doi.org/10.3390/ijms23073507
101. Ng TI, Correia I, Seagal J, DeGoey DA, Schrimpf MR, Hardee DJ, Noey EL, Kati WM (2022) Antiviral Drug Discovery for the Treatment of COVID-19 Infections. Viruses. https://doi.org/10.3390/v14050961
102. Nocentini A, Capasso C, Supuran CT (2022) Perspectives on the design and discovery of α-ketoamide inhibitors for the treatment of novel coronavirus: where do we stand and where do we go? Expert Opin Drug Discov 17:547–557
103. Ou J, Lewandowski EM, Hu Y, et al (2022) A yeast-based system to study SARS-CoV-2 M pro structure and to identify nirmatrelvir resistant mutations. bioRxiv. https://doi.org/10.1101/2022.08.06.503039

104. Ouyang J, Zaongo SD, Harypursat V, Li X, Routy J-P, Chen Y (2022) SARS-CoV-2 pre-exposure prophylaxis: A potential COVID-19 preventive strategy for high-risk populations, including healthcare workers, immunodeficient individuals, and poor vaccine responders. Front Public Health 10:945448
105. Parums DV (2022) Editorial: Current Status of Oral Antiviral Drug Treatments for SARS-CoV-2 Infection in Non-Hospitalized Patients. Med Sci Monit 28:e935952
106. Pavan M, Bassani D, Sturlese M, Moro S (2022) Bat coronaviruses related to SARS-CoV-2: what about their 3CL proteases (MPro)? J Enzyme Inhib Med Chem 37:1077–1082
107. Pavan M, Bassani D, Sturlese M, Moro S (2022) From the Wuhan-Hu-1 strain to the XD and XE variants: is targeting the SARS-CoV-2 spike protein still a pharmaceutically relevant option against COVID-19? J Enzyme Inhib Med Chem 37:1704–1714
108. Pawankar R, Thong BY-H, Tiongco-Recto M, et al (2022) Asia Pacific perspectives on the second year of the COVID-19 pandemic: A follow-up survey. Clin Exp Allergy 52:965–973
109. Peluso MJ, Anglin K, Durstenfeld MS, Martin JN, Kelly JD, Hsue PY, Henrich TJ, Deeks SG (2022) Effect of Oral Nirmatrelvir on Long COVID Symptoms: 4 Cases and Rationale for Systematic Studies. Pathog Immun 7:95–103
110. Persad G, Peek ME, Shah SK (2022) Fair Allocation of Scarce Therapies for Coronavirus Disease 2019 (COVID-19). Clin Infect Dis 75:e529–e533
111. Pesko B, Deng A, Chan JD, Neme S, Dhanireddy S, Jain R (2022) Safety, and Tolerability of Paxlovid (nirmatrelvir/ritonavir) in High Risk Patients. Clin Infect Dis. https://doi.org/10.1093/cid/ciac588
112. Phizackerley D (2022) Three more points about Paxlovid for covid-19. BMJ 377:o1397
113. Priya P, Basit A, Bandyopadhyay P (2022) A strategy to optimize the peptide-based inhibitors against different mutants of the spike protein of SARS-CoV-2. J Biomol Struct Dyn 1–12
114. Reina J, Iglesias C (2022) [Nirmatrelvir plus ritonavir (Paxlovid) a potent SARS-CoV-2 3CLpro protease inhibitor combination]. Rev Esp Quimioter 35:236–240
115. Ridgway H, Moore GJ, Mavromoustakos T, et al (2022) Discovery of a new generation of angiotensin receptor blocking drugs: Receptor mechanisms and in silico binding to enzymes relevant to SARS-CoV-2. Comput Struct Biotechnol J 20:2091–2111
116. Roberts JA, Duncan A, Cairns KA (2022) Pandora's box: Paxlovid, prescribing, pharmacists and pandemic. Am J Pharmacogenomics 52:1–4
117. S K SR, P A A, B S, Kalala KP, Pm A, Sabarathinam S (2022) Drug interaction risk between cardioprotective drugs and drugs used in treatment of COVID-19: A evidence-based review from six databases. Diabetes Metab Syndr 16:102451
118. Wang S, Gelfand JM, Calabrese C (2022) Outpatient Management of COVID-19: A Primer for the Dermatologist. Curr Dermatol Rep 1–10
119. Sakamuru S, Huang R, Xia M (2022) Use of Tox21 Screening Data to Evaluate the COVID-19 Drug Candidates for Their Potential Toxic Effects and Related Pathways. Front Pharmacol 13:935399
120. Salerno DM, Jennings DL, Lange NW, Kovac DB, Shertel T, Chen JK, Hedvat J, Scheffert J, Brown RS Jr, Pereira MR (2022) Early clinical experience with nirmatrelvir/ritonavir for the treatment of COVID-19 in solid organ transplant recipients. Am J Transplant 22:2083–2088
121. Sánchez Fabra D, Herrero Jordán T (2022) Generalized use of Nirmatrelvir plus ritonavir (Paxlovid): Raising concerns. Enferm Infecc Microbiol Clin. https://doi.org/10.1016/j.eimc.2022.04.007
122. Saravolatz LD, Depcinski S, Sharma M (2022) Molnupiravir and Nirmatrelvir-Ritonavir: Oral COVID Antiviral Drugs. Clin Infect Dis. https://doi.org/10.1093/cid/ciac180
123. Sathish JG, Bhatt S, DaSilva JK, et al (2022) Comprehensive Nonclinical Safety Assessment of Nirmatrelvir Supporting Timely Development of the SARS-COV-2 Antiviral Therapeutic, Paxlovid™. Int J Toxicol 41:276–290
124. Schöning V, Kern C, Chaccour C, Hammann F (2022) Effectiveness of Antiviral Therapy in Highly-Transmissible Variants of SARS-CoV-2: A Modeling and Simulation Study. Front Pharmacol 13:816429
125. Schwartz E (2022) Does ivermectin have a place in the treatment of mild Covid-19? New Microbes New Infect 46:100985
126. Secretan P-H, Annereau M, Kini-Matondo W, Prost B, Prudhomme J, Bournane L, Paul M, Yagoubi N, Sadou-Yayé H, Do B (2022) Unequal Behaviour between Hydrolysable Functions of Nirmatrelvir under Stress Conditions: Structural and Theoretical Approaches in Support of Preformulation Studies. Pharmaceutics. https://doi.org/10.3390/pharmaceutics14081720
127. Service RF (2022) Bad news for Paxlovid? Resistance may be coming. Science 377:138–139
128. Setz C, Große M, Auth J, Fröba M, Rauch P, Bausch A, Wright M, Schubert U (2022) Synergistic Antiviral Activity of Pamapimod and Pioglitazone against SARS-CoV-2 and Its Variants of Concern. Int J Mol Sci. https://doi.org/10.3390/ijms23126830
129. Singh AK, Singh A, Singh R, Misra A (2022) An updated practical guideline on use of molnupiravir and comparison with agents having emergency use authorization for treatment of COVID-19. Diabetes Metab Syndr 16:102396
130. Stifani BM, Madden T, Micks E, Moayedi G, Tarleton J, Benson LS (2022) Society of Family Planning Clinical Recommendations: Contraceptive Care in the Context of Pandemic Response. Contraception 113:1–12
131. Sun F, Lin Y, Wang X, Gao Y, Ye S (2022) Paxlovid in patients who are immunocompromised and hospitalised with SARS-CoV-2 infection. Lancet Infect Dis. https://doi.org/10.1016/S1473-3099(22)00430-3
132. Tang P-F, Bao S-S, Gao N-Y, Shao C-F, Xie W-F, Wu X-M, Zhao L-P, Xiao Z-X (2022) Development and Validation of a UHPLC-MS/MS Method for Quantitation of Almonertinib in Rat Plasma: Application to an in vivo Interaction Study Between Paxlovid and Almonertinib. Front Pharmacol 13:960311
133. Tanne JH (2022) Covid-19: FDA authorises pharmacists to prescribe Paxlovid. BMJ 378:o1695

134. Tarnawski AS, Ahluwalia A (2022) Endothelial cells and blood vessels are major targets for COVID-19-induced tissue injury and spreading to various organs. World J Gastroenterol 28:275–289
135. Tene L, Chodick G, Fallach N, Ansari W, Distelman-Menachem T, Maor Y (2022) Describing COVID-19 Patients During The First Two Months of Paxlovid (nirmatrelvir/ritonavir) Initiation in a Large HMO in Israel. bioRxiv. https://doi.org/10.1101/2022.05.02.22274586
136. Tolomeo M, Cavalli A, Cascio A (2022) STAT1 and Its Crucial Role in the Control of Viral Infections. Int J Mol Sci. https://doi.org/10.3390/ijms23084095
137. Traynor K (2022) Québec authorizes pharmacists to prescribe Paxlovid. Am J Health Syst Pharm. https://doi.org/10.1093/ajhp/zxac163
138. Uchikoba S, Yamada G, Tsuzuki S (2022) Methodological concerns regarding a retrospective study with real-world data on Paxlovid® in Israel. Clin Infect Dis. https://doi.org/10.1093/cid/ciac665
139. Usher AD (2022) The global COVID-19 treatment divide. Lancet 399:779–782
140. Viedma Martínez M, Gallo Pineda G, Jiménez Gallo D (2022) Practical Guide to New Treatments for SARS-CoV-2 Infection in Dermatology Patients Being Treated With Common Immunomodulators. Actas Dermosifiliogr. https://doi.org/10.1016/j.ad.2022.07.020
141. Vitiello A, Ferrara F, Auti AM, Di Domenico M, Boccellino M (2022) Advances in the Omicron variant development. J Intern Med 292:81–90
142. Vitiello A, La Porta R, Trama U, Ferrara F, Zovi A, Auti AM, Di Domenico M, Boccellino M (2022) Pandemic COVID-19, an update of current status and new therapeutic strategies. Naunyn Schmiedebergs Arch Pharmacol 395:1159–1165
143. Vuorio A, Kovanen PT, Raal F (2022) Cholesterol-lowering drugs for high-risk hypercholesterolemia patients with COVID-19 while on Paxlovid™ therapy. Future Virol. https://doi.org/10.2217/fvl-2022-0060
144. Wu C-R, Yin W-C, Jiang Y, Xu HE (2022) Structure genomics of SARS-CoV-2 and its Omicron variant: drug design templates for COVID-19. Acta Pharmacol Sin. https://doi.org/10.1038/s41401-021-00851-w
145. Wang Y, Chen X, Xiao W, Zhao D, Feng L (2022) Rapid COVID-19 rebound in a severe COVID-19 patient during 20-day course of Paxlovid. J Infect. https://doi.org/10.1016/j.jinf.2022.08.012
146. Yan G, Zhou J, Zhu H, et al (2022) The feasibility, safety, and efficacy of Paxlovid treatment in SARS-CoV-2-infected children aged 6-14 years: a cohort study. Ann Transl Med 10:619
147. Yang KS, Leeuwon SZ, Xu S, Liu WR (2022) Evolutionary and Structural Insights about Potential SARS-CoV-2 Evasion of Nirmatrelvir. J Med Chem 65:8686–8698
148. Young C, Papiro T, Greenberg JH (2022) Elevated tacrolimus levels after treatment with nirmatrelvir/ritonavir (Paxlovid) for COVID-19 infection in a child with a kidney transplant. Pediatr Nephrol. https://doi.org/10.1007/s00467-022-05712-0
149. Wang Z, Chan ECY (2022) Physiologically-Based Pharmacokinetic Modeling-Guided Dose Management of Oral Anticoagulants when Initiating Nirmatrelvir/Ritonavir (Paxlovid) for COVID-19 Treatment. Clin Pharmacol Ther 112:803–807
150. Wang Z, Yang L (2022) In the age of Omicron variant: Paxlovid raises new hopes of COVID-19 recovery. J Med Virol 94:1766–1767
151. Zhang C (2022) Fluorine in Medicinal Chemistry: In Perspective to COVID-19. ACS Omega 7:18206–18212
152. Zhu F, Ang JY (2022) COVID-19 Infection in Children: Diagnosis and Management. Curr Infect Dis Rep 24:51–62
153. Wang Y, Zhao D, Xiao W, Shi J, Chen W, Jia Q, Zhou Y, Wang R, Chen X, Feng L (2023) Paxlovid reduces the risk of Long COVID in patients six months after hospital discharge. J Med Virol 95:e29014
154. Bakos É, Temesszentandrási-Ambrus C, Özvegy-Laczka C, Gáborik Z, Sarkadi B, Telbisz Á (2023) Interactions of the Anti-SARS-CoV-2 Agents Molnupiravir and Nirmatrelvir/Paxlovid with Human Drug Transporters. Int J Mol Sci. https://doi.org/10.3390/ijms241411237
155. Yang M-J, Jiang L, Xu L, Guo S-L (2023) Association between Paxlovid and Mortality in Critically ill COVID-19 Patients Receiving Invasive Mechanical Ventilation: A Retrospective Cohort Study. Chest. https://doi.org/10.1016/j.chest.2023.07.012
156. Tan B, Sacco M, Tan H, Li K, Joyce R, Zhang X, Chen Y, Wang J (2023) Exploring diverse reactive warheads for the design of SARS-CoV-2 main protease inhibitors. Eur J Med Chem 259:115667
157. Chiu T-Y, Weng C-C, Ha SC, Tsai H-W, Koh C-C, Chen Y (2023) Management of COVID-19 Infection in a Small Bowel Transplant Recipient: A Case Report. Transplant Proc. https://doi.org/10.1016/j.transproceed.2023.05.008
158. Del Carpio-Orantes L (2023) Etiopathogenic theories about long COVID. World J Virol 12:204–208
159. Sebők S, Gyires K (2023) Long COVID and possible preventive options. Inflammopharmacology. https://doi.org/10.1007/s10787-023-01204-1
160. Lu Z, Kuang Z, Li B, Song Z, Huang L (2023) Understanding the viral shedding time of Omicron variant BA.2 infection in Shanghai: A population-based observational study. Heliyon 9:e17173
161. Tam NM, Nguyen TH, Pham MQ, Hong ND, Tung NT, Vu VV, Quang DT, Ngo ST (2023) Upgrading nirmatrelvir to inhibit SARS-CoV-2 Mpro via DeepFrag and free energy calculations. J Mol Graph Model 124:108535
162. Harris E (2023) FDA Grants Full Approval to Paxlovid, COVID-19 Antiviral Treatment. JAMA 329:2118
163. Karim F, Amardeep K, Yee A, Berson B, Cook P (2023) Mixed Warm and Cold Autoimmune Hemolytic Anemia With Concomitant Immune Thrombocytopenia Following Recent SARS-CoV-2 Infection and Ongoing Rhinovirus Infection. Cureus 15:e38509

164. Allais C, Connor CG, Do NM, et al (2023) Development of the Commercial Manufacturing Process for Nirmatrelvir in 17 Months. ACS Cent Sci 9:849–857
165. Ranard BL, Chow CC, Megjhani M, Asgari S, Park S, Vodovotz Y (2023) A mathematical model of SARS-CoV-2 immunity predicts paxlovid rebound. J Med Virol 95:e28854
166. Modi S, Kahwash R, Kissling K (2023) Case Report: tacrolimus toxicity in the setting of concurrent Paxlovid use in a heart-transplant recipient. Eur Heart J Case Rep 7:ytad193
167. Yang L, Wang Z (2023) Bench-to-bedside: Innovation of small molecule anti-SARS-CoV-2 drugs in China. Eur J Med Chem 257:115503
168. Alam F, Ananbeh O, Malik KM, Odayani AA, Hussain IB, Kaabia N, Aidaroos AA, Saudagar AKJ (2023) Towards Predicting Length of Stay and Identification of Cohort Risk Factors Using Self-Attention-Based Transformers and Association Mining: COVID-19 as a Phenotype. Diagnostics (Basel). https://doi.org/10.3390/diagnostics13101760
169. Dian Y, Meng Y, Sun Y, Deng G, Zeng F (2023) Azvudine versus Paxlovid for oral treatment of COVID-19 in Chinese patients with pre-existing comorbidities. J Infect 87:e24–e27
170. Li Y, Liu Y, Wen L, Chen H, Wang W, Tian M, Cheng Y, Xue H, Chen C (2023) Clinical efficacy analysis of paxlovid in children with hematological diseases infected with the omicron SARS-CoV-2 new variant. Front Pediatr 11:1160929
171. Park HJ, Tan ST, León TM, Jain S, Schechter R, Lo NC (2023) Predicting the public health impact of bivalent vaccines and nirmatrelvir-ritonavir against COVID-19. medRxiv. https://doi.org/10.1101/2023.05.18.23289533
172. Wang H, Chen R, Guo J, Shui L, Xiong J, Chen Y (2023) Pneumonia in newly diagnosed patients infected with the Omicron variant: a population-based study of Chinese patients in Chongqing. BMJ Open Respir Res. https://doi.org/10.1136/bmjresp-2023-001729
173. Ariel H, Ricupero A, Cooke JP (2023) Reassessing withholding of statins during Paxlovid therapy: Is it worth risking loss of the pleiotropic effects of statins and cardiovascular protection? Am J Health Syst Pharm. https://doi.org/10.1093/ajhp/zxad135
174. Ye L, Fan S, Zhao P, Wu C, Liu M, Hu S, Wang P, Wang H, Bi H (2023) Potential herb–drug interactions between anti-COVID-19 drugs and traditional Chinese medicine. Acta Pharm Sin B. https://doi.org/10.1016/j.apsb.2023.06.001
175. Tadmor T, Melamed G, Patalon T, Alapi H, Rokach L (2023) The course of patients with hairy cell leukemia during the omicron surge of the Covid-19 pandemic. Hematol Oncol. https://doi.org/10.1002/hon.3203
176. Zhang W, Li L, Zhou Z, Liu Q, Wang G, Liu D (2023) Cost-effectiveness of Paxlovid in reducing severe COVID-19 and mortality in China. Front Public Health 11:1174879
177. Perelson AS, Ribeiro RM, Phan T (2023) An explanation for SARS-CoV-2 rebound after Paxlovid treatment. medRxiv. https://doi.org/10.1101/2023.05.30.23290747
178. Chen X, Li P, Huang J, et al (2023) Discovery of novel bicyclic[3.3.0]proline peptidyl α-ketoamides as potent 3CL-protease inhibitors for SARS-CoV-2. Bioorg Med Chem Lett 90:129324
179. Salem Y, Alam Z, Shalabi MM, Hosler GA, Acharya S (2023) IgA Vasculitis Associated With COVID-19. Cureus 15:e38725
180. Wang Z, Pan Q, Ma L, et al (2023) Anthracyclines inhibit SARS-CoV-2 infection. Virus Res 334:199164
181. Smith DJ, Bi H, Hamman J, et al (2023) Potential pharmacokinetic interactions with concurrent use of herbal medicines and a ritonavir-boosted COVID-19 protease inhibitor in low and middle-income countries. Front Pharmacol 14:1210579
182. Zimmermann GW (2023) Paxlovid-Bevorratung: Apotheker wollen jetzt Geld von den Praxen. MMW Fortschr Med 165:35
183. Casey B 3rd, Vernick RC, Bahekar A, Patel D, Ncogo Alene I (2023) Ranolazine Toxicity Secondary to Paxlovid. Cureus 15:e37153
184. De SK (2023) Recent Discovery of Peptidomimetics for the Treatment of Coronavirus (COVID-19), Human coronavirus, and Enteroviruses. Curr Med Chem. https://doi.org/10.2174/0929867330666230504150758
185. Carlin AF, Clark AE, Garretson AF, Bray W, Porrachia M, Santos AT, Rana TM, Chaillon A, Smith DM (2023) Neutralizing Antibody Responses After Severe Acute Respiratory Syndrome Coronavirus 2 BA.2 and BA.2.12.1 Infection Do Not Neutralize BA.4 and BA.5 and Can Be Blunted by Nirmatrelvir/Ritonavir Treatment. Open Forum Infect Dis 10:ofad154
186. Zaidi SMH, Iskander PA, Ahmed K, Jaber F, Paz M, Khan A, Malik F, Aloysius MM (2023) A Rare Case of Paxlovid-Induced Pancreatitis. Cureus 15:e36528
187. Khan S, Fama J (2023) Acute Encephalopathy Due to Polypharmacy Interactions With the Use of Paxlovid: A Case Report. Cureus 15:e36535
188. Yang D-W, Ju M-J, Wang H, Jia Y-C, Wang X-D, Fang H, Fan J (2023) Proxalutamide for the treatment of COVID-19 rebound following Paxlovid treatment: Report of four cases and review of the literature. J Clin Lab Anal 37:e24880
189. Tang Y, Li Y, Song T (2023) Optimizing the use of nirmatrelvir/ritonavir in solid organ transplant recipients with COVID-19: A review of immunosuppressant adjustment strategies. Front Immunol 14:1150341
190. Wang R, Calderwood MS, Worby CP, Mercado JR, Kim JJ (2023) National survey study of nirmatrelvir-ritonavir prescribing processes and access barriers during the severe acute respiratory coronavirus virus 2 (SARS-CoV-2) omicron surge of the coronavirus disease 2019 (COVID-19) pandemic. Infect Control Hosp Epidemiol 1–3
191. Albrecht JM, Cooper BR, Waller JD, Presley CL, Pulsipher KJ, Rundle CW, Dellavalle RP (2023) Nirmatrelvir-ritonivir, COVID-19, and possible adverse cutaneous reactions. Dermatol Online J. https://doi.org/10.5070/D329160209
192. Zhao D, He Y, Dian Y, Meng Y, Zeng F, Deng G (2023) Elevated troponin levels predict the reduced efficacy of Paxlovid in COVID-19 patients. J Infect 87:148–150
193. de Oliveira LM, da Silva Dal Pizzol T (2023) Comment on "Tacrolimus Drug-Drug Interaction with Nirmatrelvir/Ritonavir (Paxlovid™) Managed with Phenytoin." J Med Toxicol 19:307–308

194. McCarthy MW (2023) VV116 as a potential treatment for COVID-19. Expert Opin Pharmacother 24:675–678
195. Khunte M, Kumar S, Salomon JA, Bilinski A (2023) Projected COVID-19 Mortality Reduction From Paxlovid Rollout. JAMA Health Forum 4:e230046
196. Lin CY, Cassidy AG, Li L, Prahl MK, Golan Y, Gaw SL (2023) Nirmatrelvir-Ritonavir (Paxlovid) for Mild Coronavirus Disease 2019 (COVID-19) in Pregnancy and Lactation. Obstet Gynecol 141:957–960
197. Kim Y, Ryu JY, Kim HU, Lee SY (2023) Computational prediction of interactions between Paxlovid and prescription drugs. Proc Natl Acad Sci U S A 120:e2221857120
198. Moghadasi SA, Biswas RG, Harki DA, Harris RS (2023) Rapid resistance profiling of SARS-CoV-2 protease inhibitors. bioRxiv. https://doi.org/10.1101/2023.02.25.530000
199. Pavan M, Moro S (2023) Lessons Learnt from COVID-19: Computational Strategies for Facing Present and Future Pandemics. Int J Mol Sci. https://doi.org/10.3390/ijms24054401
200. Banerjee S, Banerjee D, Singh A, Kumar S, Pooja D, Ram V, Kulhari H, Saharan VA (2023) A Clinical Insight on New Discovered Molecules and Repurposed Drugs for the Treatment of COVID-19. Vaccines (Basel). https://doi.org/10.3390/vaccines11020332
201. Belden KA, Yeager S, Schulte J, Cantarin MPM, Moss S, Royer T, Coppock D (2023) "Saving lives with nirmatrelvir/ritonavir one transplant patient at a time." Transpl Infect Dis 25:e14037
202. Michael S, Heilbronner R, Lloyd CM, Levitin HW (2023) Paxlovid-Induced Tacrolimus Toxicity in the Treatment of COVID-19: A Case Report. Cureus 15:e35489
203. Srisubat A, Thanasitthichai S, Kongsaengdao S, Maneeton N, Maneeton B, Akksilp S (2023) Effectiveness of Favipiravir monotherapy in the treatment of COVID-19: real world data analysis from Thailand. Lancet Reg Health Southeast Asia 11:100166
204. Li J, Wang Y, Solanki K, Atre R, Lavrijsen M, Pan Q, Baig MS, Li P (2023) Nirmatrelvir exerts distinct antiviral potency against different human coronaviruses. Antiviral Res 211:105555
205. Chen MJ, Cheema R, Hoyt-Austin A, Agnoli A, Kuhn-Riordon K, Kair LR (2023) Vaccination and treatment options for SARS-CoV2 infection affecting lactation and breastfeeding. Semin Fetal Neonatal Med 28:101425
206. Culas R, Nath S, Nath S (2023) Safely Prescribing Nirmatrelvir and Ritonavir-Avoiding Drug-Drug Interactions. JAMA Intern Med 183:362–363
207. Cvancara DJ, Baertsch HC, Lehmann AE, Humphreys IM, Farrell NF, Marshall TB, Bhatt NK, Abuzeid WM, Jafari A (2023) Postmarketing Reporting of Paxlovid-Related Dysgeusia: A Real-World Pharmacovigilance Study. Otolaryngol Head Neck Surg 169:55–61
208. Kale A, Shelke V, Dagar N, Anders H-J, Gaikwad AB (2023) How to use COVID-19 antiviral drugs in patients with chronic kidney disease. Front Pharmacol 14:1053814
209. Zhong W, Yang X, Jiang X, et al (2022) Factors associated with prolonged viral shedding in older patients infected with Omicron BA.2.2. Front Public Health 10:1087800
210. Chen M, Guo T, Zhou Z, Chen D, Zhou H (2022) SARS-CoV-2 Omicron BA.5 infection recurrence after Paxlovid treatment: The first case report in Hunan Province. Zhong Nan Da Xue Xue Bao Yi Xue Ban 47:1775–1780
211. Boretti A (2023) On the choice of Molnupiravir and Paxlovid as the only antivirals permitted for COVID-19 infection in Australia. Clin Exp Med 1–2
212. Harris E (2023) Paxlovid Alternative Is Similarly Effective. JAMA 329:622–623
213. Kincaid JRA, Caravez JC, Iyer KS, Kavthe RD, Fleck N, Aue DH, Lipshutz BH (2022) A sustainable synthesis of the SARS-CoV-2 Mpro inhibitor nirmatrelvir, the active ingredient in Paxlovid. Commun Chem 5:156
214. Chourasia P, Maringanti BS, Edwards-Fligner M, Gangu K, Bobba A, Sheikh AB, Shekhar R (2023) Paxlovid (Nirmatrelvir and Ritonavir) Use in Pregnant and Lactating Woman: Current Evidence and Practice Guidelines-A Scoping Review. Vaccines (Basel). https://doi.org/10.3390/vaccines11010107
215. Degirmenci HB, Oh J, Bays AM, Thomason JL, Liew JW (2022) Public Interest in COVID-19 Therapeutics for High-Risk Populations During the Omicron Era: A Google Trends Analysis. Cureus 14:e32684
216. Lan S, Neilsen G, Slack RL, et al (2023) Nirmatrelvir Resistance in SARS-CoV-2 Omicron_BA.1 and WA1 Replicons and Escape Strategies. bioRxiv. https://doi.org/10.1101/2022.12.31.522389
217. Ganipisetti VM, Bollimunta P, Maringanti S (2023) Paxlovid-Induced Symptomatic Bradycardia and Syncope. Cureus 15:e33831
218. Chinese Thoracic Society, National Center for Respiratory Medicine (2023) [Expert consensus on the diagnosis and treatment of SARS-CoV-2-associated cough]. Zhonghua Jie He He Hu Xi Za Zhi 46:E002
219. Kozlov M (2023) COVID drug Paxlovid was hailed as a game-changer. What happened? Nature 613:224–225
220. Miljković MD, Prasad V (2023) Paxlovid: A Regulatory Gamble. Am J Med 136:336–338
221. Focosi D, McConnell S, Shoham S, Casadevall A, Maggi F, Antonelli G (2023) Nirmatrelvir and COVID-19: development, pharmacokinetics, clinical efficacy, resistance, relapse, and pharmacoeconomics. Int J Antimicrob Agents 61:106708
222. Manóchio C, Torres-Loureiro S, Scudeler MM, Miwa B, Souza-Santos FC, Rodrigues-Soares F (2023) Theranostics for COVID-19 Antiviral Drugs: Prospects and Challenges for Worldwide Precision/Personalized Medicine. OMICS 27:6–14
223. Liu C-I, Goh KK, Chen C-H (2022) Neutropenia after the coadministration of clozapine and nirmatrelvir-ritonavir in a patient with SARS-CoV-2 infection: A case report with a literature review. Front Psychiatry 13:1096006
224. Melton TC, Hawkins BK (2022) A Community Pharmacist's Guide to Oral COVID-19 Antivirals. Innov Pharm. https://doi.org/10.24926/iip.v13i3.4961

225. Yang KS, Blankenship LR, Kuo S-TA, Sheng YJ, Li P, Fierke CA, Russell DH, Yan X, Xu S, Liu WR (2023) A Novel Y-Shaped, S-O-N-O-S-Bridged Cross-Link between Three Residues C22, C44, and K61 Is Frequently Observed in the SARS-CoV-2 Main Protease. ACS Chem Biol 18:449–455
226. Haque OI, Mahar S, Hussain S, Sloane P (2023) Pharmacokinetic interaction between verapamil and ritonavir-boosted nirmatrelvir: implications for the management of COVID-19 in patients with hypertension. BMJ Case Rep. https://doi.org/10.1136/bcr-2022-252677
227. Mangurian C (2023) Getting Treated for COVID-19 Shouldn't Be This Difficult. JAMA 329:123–124
228. Esposito S, Autore G, Argentiero A, Ramundo G, Perrone S, Principi N (2022) Update on COVID-19 Therapy in Pediatric Age. Pharmaceuticals . https://doi.org/10.3390/ph15121512
229. Anwar K, Nguyen L, Nagasaka M, Ou S-HI, Chan A (2023) Overview of Drug-Drug Interactions Between Ritonavir-Boosted Nirmatrelvir (Paxlovid) and Targeted Therapy and Supportive Care for Lung Cancer. JTO Clin Res Rep 4:100452
230. Wang Y, Zhao D, Liu X, Chen X, Xiao W, Feng L (2023) Early administration of Paxlovid reduces the viral elimination time in patients infected with SARS-CoV-2 Omicron variants. J Med Virol 95:e28443
231. Bose-Brill S, Hirabayashi K, Pajor NM, et al (2022) Pediatric Nirmatrelvir/Ritonavir Prescribing Patterns During the COVID-19 Pandemic. medRxiv. https://doi.org/10.1101/2022.12.23.22283868
232. Kwon E-J, Yun G-A, Park S, Kim S, Chae D-W, Park HS, Lee T, Jeong JC (2022) Treatment of acute tacrolimus toxicity with phenytoin after Paxlovid (nirmatrelvir/ritonavir) administration in a kidney transplant recipient. Kidney Res Clin Pract 41:768–770
233. Philipponnet C, Aniort J, Atenza A, Heng A-E, Souweine B (2022) Carfilzomib Induced Microangiopathy due to Accumulation With Paxlovid. Kidney Int Rep 7:2746–2749
234. Kuehn BM (2022) Inequity in Paxlovid Prescribing. JAMA 328:2203–2204
235. Caravez JC, Iyer KS, Kavthe RD, Kincaid JRA, Lipshutz BH (2022) A 1-Pot Synthesis of the SARS-CoV-2 Mpro Inhibitor Nirmatrelvir, the Key Ingredient in Paxlovid. Org Lett 24:9049–9053
236. Algar-Lizana S, Bonache MÁ, González-Muñiz R (2023) SARS-CoV-2 main protease inhibitors: What is moving in the field of peptides and peptidomimetics? J Pept Sci 29:e3467
237. Alteri C, Fox V, Scutari R, et al (2022) A proof-of-concept study on the genomic evolution of Sars-Cov-2 in molnupiravir-treated, paxlovid-treated and drug-naïve patients. Commun Biol 5:1376
238. Carr TF, Fajt ML, Kraft M, Phipatanakul W, Szefler SJ, Zeki AA, Peden DB, White SR, Precision Interventions for Severe and/or Exacerbation-Prone Asthma Network Research Group (2023) Treating asthma in the time of COVID. J Allergy Clin Immunol 151:809–817
239. Sindelar M, McCabe D, Carroll E (2023) Tacrolimus Drug-Drug Interaction with Nirmatrelvir/Ritonavir (Paxlovid™) Managed with Phenytoin. J Med Toxicol 19:45–48
240. Martins V, Fazal L, Oganesian A, Shah A, Stow J, Walton H, Wilsher N (2022) A commentary on the use of pharmacoenhancers in the pharmaceutical industry and the implication for DMPK drug discovery strategies. Xenobiotica 52:786–796
241. Park JJ, Lee J, Seo YB, Na SH (2022) Nirmatrelvir/Ritonavir Prescription Rate and Outcomes in Coronavirus Disease 2019: A Single Center Study. Infect Chemother 54:757–764
242. Majerová T, Konvalinka J (2022) Viral proteases as therapeutic targets. Mol Aspects Med 88:101159
243. Iacobucci G (2022) Covid-19: "Grotesque inequity" that only a quarter of paxlovid courses go to poorer countries. BMJ 379:o2795
244. Zhang L, Zhang S, Han J, Yi Y, Zhou H, Li J (2022) Paxlovid administration in elderly patient with COVID-19 caused by Omicron BA.2.0: A case report. Medicine 101:e31361
245. Busse K, El-Alfy AT (2022) Paxlovid (nirmatrelvir/ritonavir). WMJ 121:P3
246. Tavazzi L (2022) Clinical research methodology process: what has changed with COVID-19? Eur Heart J Suppl 24:I175–I180
247. Zizioli D, Ferretti S, Mignani L, Castelli F, Tiecco G, Zanella I, Quiros-Roldan E (2023) Developmental safety of nirmatrelvir in zebrafish (Danio rerio) embryos. Birth Defects Res 115:430–440
248. Dawood AA (2023) The efficacy of Paxlovid against COVID-19 is the result of the tight molecular docking between Mpro and antiviral drugs (nirmatrelvir and ritonavir). Adv Med Sci 68:1–9
249. Kong K, Chang Y, Qiao H, et al (2022) Paxlovid accelerates cartilage degeneration and senescence through activating endoplasmic reticulum stress and interfering redox homeostasis. J Transl Med 20:549
250. Cokley JA, Gidal BE, Keller JA, Vossler DG, Reviewed and approved by the AES Treatments Committee and Council on Clinical Activities (2022) PaxlovidTM Information From FDA and Guidance for AES Members. Epilepsy Curr 22:201–204
251. Cox RM, Lieber CM, Wolf JD, et al (2022) Paxlovid-like nirmatrelvir/ritonavir fails to block SARS-CoV-2 transmission in ferrets. bioRxiv. https://doi.org/10.1101/2022.11.20.517271
252. Brault A, Tran-Kiem C, Couteaux C, Olié V, Paireau J, Yazdanpanah Y, Ghosn J, Martin-Blondel G, Bosetti P, Cauchemez S (2023) Modelling the end of a Zero-COVID strategy using nirmatrelvir/ritonavir, vaccination and NPIs in Wallis and Futuna. Lancet Reg Health West Pac 30:100634
253. Metzendorf M-I, Weibel S, Reis S, McDonald S (2023) Pragmatic and open science-based solution to a current problem in the reporting of living systematic reviews. BMJ Evid Based Med 28:267–272
254. Cadley SM, Sethi A, Knorr JP (2022) CYP induction to reverse tacrolimus toxicity resulting from concomitant Paxlovid use. Transpl Infect Dis 24:e13982

255. Stawiarski K, Avery R, Strout S, Umapathi P (2023) Risks of paxlovid in a heart transplant recipient. J Heart Lung Transplant 42:30–32
256. Moezzi MS (2022) Comprehensive in silico screening of flavonoids against SARS-CoV-2 main protease. J Biomol Struct Dyn 1–14
257. Chollet V, Manuel O, De Vallière S (2022) [COVID-19, how to treat the disease during Fall 2022?]. Rev Med Suisse 18:2077–2081
258. Sasi VM, Ullrich S, Ton J, Fry SE, Johansen-Leete J, Payne RJ, Nitsche C, Jackson CJ (2022) Predicting Antiviral Resistance Mutations in SARS-CoV-2 Main Protease with Computational and Experimental Screening. Biochemistry 61:2495–2505
259. Shi S, Dong N, Ding Y, et al (2022) [COVID-19 treated with oral Nirmatrelvir-Ritonavir in 3 children]. Zhonghua Er Ke Za Zhi 60:1168–1171
260. Bruchelt G, Treuner J, Schmidt K (2022) Proposal for the use of an inhalation drug containing 2-5 oligoadenylates for treatment of COVID-19. Med Hypotheses 168:110969
261. Sindelar M, McCabe D, Carroll E (2023) Response to Comment on "Tacrolimus Drug-Drug Interaction with Nirmatrelvir/Ritonavir (Paxlovid™) Managed with Phenytoin." J Med Toxicol 19:309
262. Zhu B, Wei X, Narasimhan H, et al (2023) Inhibition of the mitochondrial pyruvate carrier simultaneously mitigates hyperinflammation and hyperglycemia in COVID-19. Sci Immunol 8:eadf0348
263. Wang Y, Zhao D, Chen X, Liu X, Xiao W, Feng L (2023) The effect of nirmatrelvir-ritonavir on viral clearance and length of hospital stay in patients infected with SARS-CoV-2 omicron variants. Influenza Other Respi Viruses 17:e13095
264. Madan A, Garg M, Satija G, et al (2023) SAR Based Review on Diverse Heterocyclic Compounds with Various Potential Molecular Targets in the Fight against Covid-19: A Medicinal Chemist Perspective. Curr Top Med Chem. https://doi.org/10.2174/1568026623666230126104156
265. Bloch EM, Focosi D, Shoham S, et al (2023) Guidance on the Use of Convalescent Plasma to Treat Immunocompromised Patients With Coronavirus Disease 2019. Clin Infect Dis 76:2018–2024
266. Zhong L, Zhao Z, Peng X, Zou J, Yang S (2022) Recent advances in small-molecular therapeutics for COVID-19. Precis Clin Med 5:bac024
267. Martens-Lobenhoffer J, Böger CR, Kielstein J, Bode-Böger SM (2022) Simultaneous quantification of nirmatrelvir and ritonavir by LC-MS/MS in patients treated for COVID-19. J Chromatogr B Analyt Technol Biomed Life Sci 1212:123510
268. Bakacs T, Chumakov K, Safadi R, Kovesdi I (2022) Editorial: Fighting fire with fire: Using non-pathogenic viruses to control unrelated infections. Front Immunol 13:1046851
269. Moreira Pinheiro LB, Tao S, Culbertson E, Lima Barros de Araujo G, Billinge SJL, Ferreira FF (2022) Evaluation of the polymorphic forms of ritonavir and lopinavir in raw materials and co-milled systems. Int J Pharm 628:122329
270. Lingscheid T, Kinzig M, Krüger A, et al (2022) Pharmacokinetics of Nirmatrelvir and Ritonavir in COVID-19 Patients with End-Stage Renal Disease on Intermittent Hemodialysis. Antimicrob Agents Chemother 66:e0122922
271. Shah A, Nasrullah A, Butt MA, Young M (2022) Paxlovid with Caution: Novel Case of Paxlovid-Induced Tacrolimus Toxicity in a Cardiac Transplant Patient. Eur J Case Rep Intern Med 9:003528
272. Boehmer TK, Koumans EH, Skillen EL, et al (2022) Racial and Ethnic Disparities in Outpatient Treatment of COVID-19 - United States, January-July 2022. MMWR Morb Mortal Wkly Rep 71:1359–1365
273. Chang CT, Ong SY, Lim XJ, Chew LS, Rajan P (2022) Managing nirmatrelvir/ritonavir during COVID-19: pharmacists' experiences from the Perak state of Malaysia. J Pharm Policy Pract 15:70
274. Sullivan M, Perrine CG, Kelleher J, Kanwar O, Kuwabara S, Bennett K, Jackson BR, Patel P, Pennini ME (2022) Notes From the Field: Dispensing of Oral Antiviral Drugs for Treatment of COVID-19 by Zip Code-Level Social Vulnerability - United States, December 23, 2021-August 28, 2022. MMWR Morb Mortal Wkly Rep 71:1384–1385
275. Brady DK, Gurijala AR, Huang L, Hussain AA, Lingan AL, Pembridge OG, Ratangee BA, Sealy TT, Vallone KT, Clements TP (2022) A guide to COVID-19 antiviral therapeutics: a summary and perspective of the antiviral weapons against SARS-CoV-2 infection. FEBS J. https://doi.org/10.1111/febs.16662
276. Noske GD, de Souza Silva E, de Godoy MO, Dolci I, Fernandes RS, Guido RVC, Sjö P, Oliva G, Godoy AS (2023) Structural basis of nirmatrelvir and ensitrelvir activity against naturally occurring polymorphisms of the SARS-CoV-2 main protease. J Biol Chem 299:103004
277. Liang Y, Fang D, Gao X, Deng X, Chen N, Wu J, Zeng M, Luo M (2023) Circulating microRNAs as emerging regulators of COVID-19. Theranostics 13:125–147
278. Hsiao K, Zegzouti H, Goueli S (2022) High throughput bioluminescent assay to characterize and monitor the activity of SARS-CoV-2 methyltransferases. PLoS One 17:e0274343
279. Jochmans D, Liu C, Donckers K, et al (2023) The Substitutions L50F, E166A, and L167F in SARS-CoV-2 3CLpro Are Selected by a Protease Inhibitor In Vitro and Confer Resistance To Nirmatrelvir. MBio 14:e0281522
280. Parigger L, Krassnigg A, Schopper T, Singh A, Tappler K, Köchl K, Hetmann M, Gruber K, Steinkellner G, Gruber CC (2022) Recent changes in the mutational dynamics of the SARS-CoV-2 main protease substantiate the danger of emerging resistance to antiviral drugs. Front Med 9:1061142
281. Tan B, Joyce R, Tan H, Hu Y, Wang J (2023) SARS-CoV-2 Main Protease Drug Design, Assay Development, and Drug Resistance Studies. Acc Chem Res 56:157–168
282. Ghosh AK, Yadav M (2023) Synthesis of optically active SARS-CoV-2 Mpro inhibitor drug nirmatrelvir (Paxlovid): an approved treatment of COVID-19. Org Biomol Chem 21:5768–5774

283. Wahid M, Jawed A, Mandal RK, et al (2023) Role of available COVID-19 vaccines in reducing deaths and perspective for next generation vaccines and therapies to counter emerging viral variants: an update. Minerva Med. https://doi.org/10.23736/S0026-4806.23.08509-9
284. Kieck D, Mahalick L, Vo TT (2023) Medication-Related Problems Identified and Addressed by Pharmacists Dispensing COVID-19 Antivirals at a Community Pharmacy. Pharmacy (Basel). https://doi.org/10.3390/pharmacy11030087
285. Sagawa K, Lin J, Jaini R, Di L (2023) Physiologically-Based Pharmacokinetic Modeling of PAXLOVID™ with First-Order Absorption Kinetics. Pharm Res 1–12
286. Clayton J, de Oliveira VM, Ibraham MF, Sun X, Mahinthichaichan P, Shen M, Hilgenfeld R, Shen J (2023) An Integrative Approach to Dissect the Drug Resistance Mechanism of the H172Y Mutation of SARS-CoV-2 Main Protease. bioRxiv. https://doi.org/10.1101/2022.07.31.502215
287. Clayton J, de Oliveira VM, Ibrahim MF, Sun X, Mahinthichaichan P, Shen M, Hilgenfeld R, Shen J (2023) Integrative Approach to Dissect the Drug Resistance Mechanism of the H172Y Mutation of SARS-CoV-2 Main Protease. J Chem Inf Model 63:3521–3533
288. Adams AJ, Eid DD (2023) Federal pharmacist Paxlovid prescribing authority: A model policy or impediment to optimal care? Explor Res Clin Soc Pharm 9:100244
289. Van Oers TJ, Piercey A, Belovodskiy A, Reiz B, Donnelly BL, Vuong W, Lemieux MJ, Nieman JA, Auclair K, Vederas JC (2023) Deuteration for Metabolic Stabilization of SARS-CoV-2 Inhibitors GC373 and Nirmatrelvir. Org Lett. https://doi.org/10.1021/acs.orglett.3c02140
290. Cao Z, Gao W, Bao H, et al (2023) VV116 versus Nirmatrelvir-Ritonavir for Oral Treatment of Covid-19. N Engl J Med 388:406–417
291. Larkin HD (2022) Paxlovid Drug Interaction Screening Checklist Updated. JAMA 328:1290
292. Cai H, Yan J, Wang J, Che X, Mou S (2022) Efficacy of Paxlovid in patients with acute kidney injury who developed COVID-19. J Infect 85:702–769
293. Muse O, Patell R, Lee M, Lech T, Guirguis M, Dodge L, Zwicker JI (2022) Impact of Paxlovid on international normalized ratio among patients on chronic warfarin therapy. Blood 140:2757–2759
294. Aydogdu S, Hatipoglu A (2022) Electronic Structures and Reactivities of COVID-19 Drugs: A DFT Study. Acta Chim Slov 69:647–656
295. Shin SW, Cho IH (2023) Panax ginseng as a potential therapeutic for neurological disorders associated with COVID-19; Toward targeting inflammasome. J Ginseng Res 47:23–32
296. Chavda VP, Vuppu S, Mishra T, Kamaraj S, Patel AB, Sharma N, Chen Z-S (2022) Recent review of COVID-19 management: diagnosis, treatment and vaccination. Pharmacol Rep 74:1120–1148
297. Galloway S, Taunton C, Matysek R, Hempenstall A (2022) Seeking to improve access to COVID-19 therapeutics in the remote Torres and Cape communities of Far North Queensland during the first COVID-19 omicron outbreak. Rural Remote Health 22:7657
298. Jiang Y, Rubin L, Zhou Z, Zhang H, Su Q, Hou S-T, Lazarovici P, Zheng W (2022) Pharmacological therapies and drug development targeting SARS-CoV-2 infection. Cytokine Growth Factor Rev 68:13–24
299. Zhong W, Jiang X, Yang X, et al (2022) The efficacy of paxlovid in elderly patients infected with SARS-CoV-2 omicron variants: Results of a non-randomized clinical trial. Front Med 9:980002
300. Hashash JG, Desai A, Kochhar GS, Farraye FA (2023) Efficacy of Paxlovid and Lagevrio for COVID-19 Infection in Patients With Inflammatory Bowel Disease: A Propensity-Matched Study. Clin Gastroenterol Hepatol 21:841–843.e4
301. Nene RV, Navarro MR, Tomaszewski CA, Lafree A (2022) Experience Using Paxlovid for Patients With Coronavirus Disease 2019 in a Resource-Limited Emergency Department. Ann Emerg Med 80:382–383
302. De SK (2023) Novel Proline Derivatives for Treating COVID-19. Curr Med Chem 30:1458–1461
303. Pepperrell T, Ellis L, Wang J, Hill A (2022) Barriers to Worldwide Access for Paxlovid, a New Treatment for COVID-19. Open Forum Infect Dis 9:ofac174
304. Traynor K (2022) Paxlovid Prescribing Requires Pharmacy Workload Assessments. Am J Health Syst Pharm 79:1975–1976
305. Parums DV (2022) Editorial: Rebound COVID-19 and Cessation of Antiviral Treatment for SARS-CoV-2 with Paxlovid and Molnupiravir. Med Sci Monit 28:e938532
306. Calò LA, Sgarabotto L, Stefanelli LF, Di Vico V, Davis PA (2023) COVID 19, Paxlovid and the lesson from rare genetic diseases with naturally occurring protection from SARS-CoV-2 infection. J Nephrol 36:925–927
307. Charness ME, Gupta K, Stack G, Strymish J, Adams E, Lindy DC, Mohri H, Ho DD (2022) Rebound of SARS-CoV-2 Infection after Nirmatrelvir-Ritonavir Treatment. N Engl J Med 387:1045–1047
308. Anderson AS, Caubel P, Rusnak JM, EPIC-HR Trial Investigators (2022) Nirmatrelvir-Ritonavir and Viral Load Rebound in Covid-19. N Engl J Med 387:1047–1049
309. Devresse A, Sébastien Briol, De Greef J, et al (2022) Safety, Efficacy, and Relapse of Nirmatrelvir-Ritonavir in Kidney Transplant Recipients Infected With SARS-CoV-2. Kidney Int Rep 7:2356–2363
310. Ranganath N, O'Horo JC, Challener DW, Tulledge-Scheitel SM, Pike ML, O'Brien M, Razonable RR, Shah A (2023) Rebound Phenomenon After Nirmatrelvir/Ritonavir Treatment of Coronavirus Disease 2019 (COVID-19) in High-Risk Persons. Clin Infect Dis 76:e537–e539

311. Siberry GK, Mofenson LM, Calmy A, Reddy UM, Abrams EJ (2022) Use of Ritonavir-Boosted Nirmatrelvir in Pregnancy. Clin Infect Dis 75:2279–2281
312. Vuorio A, Kovanen PT, Raal F (2022) Statin Needs to be Continued During Paxlovid Therapy in COVID-19. Clin Infect Dis 75:2281–2282
313. Shao J, Fan R, Hu J, et al (2022) Clinical Progression and Outcome of Hospitalized Patients Infected with SARS-CoV-2 Omicron Variant in Shanghai, China. Vaccines (Basel). https://doi.org/10.3390/vaccines10091409
314. Schreiber A, Ambrosy B, Planz O, Schloer S, Rescher U, Ludwig S (2022) The MEK1/2 Inhibitor ATR-002 (Zapnometinib) Synergistically Potentiates the Antiviral Effect of Direct-Acting Anti-SARS-CoV-2 Drugs. Pharmaceutics. https://doi.org/10.3390/pharmaceutics14091776
315. Hu Y, Lewandowski EM, Tan H, et al (2022) Naturally occurring mutations of SARS-CoV-2 main protease confer drug resistance to nirmatrelvir. bioRxiv. https://doi.org/10.1101/2022.06.28.497978
316. Razonable RR, O'Horo JC, Hanson SN, et al (2022) Comparable Outcomes for Bebtelovimab and Ritonavir-Boosted Nirmatrelvir Treatment in High-Risk Patients With Coronavirus Disease-2019 During Severe Acute Respiratory Syndrome Coronavirus 2 BA.2 Omicron Epoch. J Infect Dis 226:1683–1687
317. Khan MM, Goh Y-W, Ahmad N, Siddique MM (2022) Understanding and combating COVID-19 using the biology and chemistry of SARS-CoV-2. Bioprocess Biosyst Eng 45:1753–1769
318. Wagoner J, Herring S, Hsiang T-Y, et al (2022) Combinations of Host- and Virus-Targeting Antiviral Drugs Confer Synergistic Suppression of SARS-CoV-2. Microbiol Spectr 10:e0333122
319. Chen W, Liang B, Wu X, Li L, Wang C, Xing D (2023) Advances and challenges in using nirmatrelvir and its derivatives against SARS-CoV-2 infection. J Pharm Anal 13:255–261
320. Liu X, Shi J, Wang D, Su Y, Xing Z, Sun F, Chen F (2023) Therapeutic Polypeptides and Peptidomimetics: Powerful Tools for COVID-19 Treatment. Clin Drug Investig 43:13–22
321. Hahn F, Wangen C, Häge S, Herrmann L, Herrmann A, Tsogoeva SB, Marschall M (2022) The Trimeric Artesunate Analog TF27, a Broadly Acting Anti-Infective Model Drug, Exerts Pronounced Anti-SARS-CoV-2 Activity Spanning Variants and Host Cell Types. Pharmaceutics. https://doi.org/10.3390/pharmaceutics15010115
322. Halder SK, Sultana I, Shuvo MN, Shil A, Himel MK, Hasan MA, Shawan MMAK (2023) In Silico Identification and Analysis of Potentially Bioactive Antiviral Phytochemicals against SARS-CoV-2: A Molecular Docking and Dynamics Simulation Approach. Biomed Res Int 2023:5469258
323. Gross R, Thaweethai T, Rosenzweig EB, et al (2023) Researching COVID to enhance recovery (RECOVER) pediatric study protocol: Rationale, objectives and design. medRxiv. https://doi.org/10.1101/2023.04.27.23289228
324. Portman DB, Scolese CJ (2023) Facilitating oral COVID-19 therapy utilization through a pharmacy consult service. J Am Pharm Assoc 63:1237–1241
325. Rungruangmaitree R, Phoochaijaroen S, Chimprasit A, Saparpakorn P, Pootanakit K, Tanramluk D (2023) Structural analysis of the coronavirus main protease for the design of pan-variant inhibitors. Sci Rep 13:7055
326. Chan M, Linn MMN, O'Hagan T, et al (2023) Persistent SARS-CoV-2 PCR Positivity Despite Anti-viral Treatment in Immunodeficient Patients. J Clin Immunol 43:1083–1092
327. Loos NHC, Beijnen JH, Schinkel AH (2023) The inhibitory and inducing effects of ritonavir on hepatic and intestinal CYP3A and other drug-handling proteins. Biomed Pharmacother 162:114636
328. Bihan K, Lipszyc L, Lemaitre F, Dautriche A, Fédrizzi S, Atzenhoffer M, Vitores A, Page A, Lebrun-Vignes B (2023) Nirmatrelvir/ritonavir (Paxlovid®): French pharmacovigilance survey 2022. Therapie. https://doi.org/10.1016/j.therap.2023.03.001
329. Paciaroni A, Libera V, Ripanti F, et al (2023) Stabilization of the Dimeric State of SARS-CoV-2 Main Protease by GC376 and Nirmatrelvir. Int J Mol Sci. https://doi.org/10.3390/ijms24076062
330. Saramago LC, Santana MV, Gomes BF, et al (2023) AI-Driven Discovery of SARS-CoV-2 Main Protease Fragment-like Inhibitors with Antiviral Activity In Vitro. J Chem Inf Model 63:2866–2880
331. Basit SA, Qureshi R, Musleh S, Guler R, Rahman MS, Biswas KH, Alam T (2023) COVID-19Base v3: Update of the knowledgebase for drugs and biomedical entities linked to COVID-19. Front Public Health 11:1125917
332. England C, TrejoMartinez J, PerezSanchez P, Karki U, Xu J (2023) Plants as Biofactories for Therapeutic Proteins and Antiviral Compounds to Combat COVID-19. Life. https://doi.org/10.3390/life13030617
333. Amin T, Kesselheim AS (2022) A Global Intellectual Property Waiver is Still Needed to Address the Inequities of COVID-19 and Future Pandemic Preparedness. Inquiry 59:469580221124821
334. Shi HJ, Yang J, Eom JS, et al (2023) Clinical Characteristics and Risk Factors for Mortality in Critical COVID-19 Patients Aged 50 Years or Younger During Omicron Wave in Korea: Comparison With Patients Older Than 50 Years of Age. J Korean Med Sci 38:e217
335. Ibeh RC, Ikechukwu GC, Ukweni CJ, et al (2023) Computational studies of potential antiviral compounds from some selected Nigerian medicinal plants against SARS-CoV-2 proteins. Inform Med Unlocked 38:101230
336. Green ACA, Curtis HJ, Higgins R, et al (2023) Trends, variation, and clinical characteristics of recipients of antiviral drugs and neutralising monoclonal antibodies for covid-19 in community settings: retrospective, descriptive cohort study of 23.4 million people in OpenSAFELY. BMJ Med 2:e000276

337. Ip JD, Wing-Ho Chu A, Chan W-M, Cheuk-Ying Leung R, Umer Abdullah SM, Sun Y, Kai-Wang To K (2023) Global prevalence of SARS-CoV-2 3CL protease mutations associated with nirmatrelvir or ensitrelvir resistance. EBioMedicine 91:104559
338. Grandvuillemin A, Rocher F, Valnet-Rabier MB, Drici M-D, Dautriche A, French Pharmacovigilance Network (2023) Pharmacovigilance follow-up of patients in the context of the COVID-19 pandemic. Therapie. https://doi.org/10.1016/j.therap.2023.01.004
339. Dechtman I-D, Ankory R, Sokolinsky K, Krasner E, Weiss L, Gal Y (2023) Clinically Evaluated COVID-19 Drugs with Therapeutic Potential for Biological Warfare Agents. Microorganisms. https://doi.org/10.3390/microorganisms11061577
340. Kiouri DP, Ntallis C, Kelaidonis K, et al (2023) Network-Based Prediction of Side Effects of Repurposed Antihypertensive Sartans against COVID-19 via Proteome and Drug-Target Interactomes. Proteomes. https://doi.org/10.3390/proteomes11020021
341. Uzuner U, Akkus E, Kocak A, Çelik Uzuner S (2023) Exploring epigenetic drugs as potential inhibitors of SARS-CoV-2 main protease: a docking and MD simulation study. J Biomol Struct Dyn 1–12
342. Puhl AC, Godoy AS, Noske GD, Nakamura AM, Gawriljuk VO, Fernandes RS, Oliva G, Ekins S (2023) Discovery of PLpro and Mpro Inhibitors for SARS-CoV-2. ACS Omega 8:22603–22612
343. Qu Y, Su C, Xiang Z, Wang Y, Han J, Pan J, Shen Z (2023) Population pharmacokinetic modeling and simulation for nirmatrelvir exposure assessment in Chinese older patients with COVID-19 infection. Eur J Pharm Sci 189:106535
344. Gerhart J, Draica F, Benigno M, Atkinson J, Reimbaeva M, Francis D, Baillon-Plot N, Sidhu GS, Damle BD (2023) Real-World Evidence of the Top 100 Prescribed Drugs in the USA and Their Potential for Drug Interactions with Nirmatrelvir; Ritonavir. AAPS J 25:73
345. Lee CYS, Suzuki JB (2023) COVID-19: Variants, Immunity, and Therapeutics for Non-Hospitalized Patients. Biomedicines. https://doi.org/10.3390/biomedicines11072055
346. Zhang W, Yang Z, Zhou F, Wei Y, Ma X (2022) Network Pharmacology and Bioinformatics Analysis Identifies Potential Therapeutic Targets of Paxlovid Against LUAD/COVID-19. Front Endocrinol 13:935906
347. Madariaga-Mazón A, Naveja JJ, Becerra A, Alberto Campillo-Balderas J, Hernández-Morales R, Jácome R, Lazcano A, Martinez-Mayorga K (2022) Subtle structural differences of nucleotide analogs may impact SARS-CoV-2 RNA-dependent RNA polymerase and exoribonuclease activity. Comput Struct Biotechnol J 20:5181–5192
348. Blair HA (2023) Nirmatrelvir plus ritonavir in COVID-19: a profile of its use. Drugs Ther Perspect 39:41–47
349. Haddad F, Dokmak G, Karaman R (2022) A Comprehensive Review on the Efficacy of Several Pharmacologic Agents for the Treatment of COVID-19. Life. https://doi.org/10.3390/life12111758
350. Reis S, Metzendorf M-I, Kuehn R, Popp M, Gagyor I, Kranke P, Meybohm P, Skoetz N, Weibel S (2022) Nirmatrelvir combined with ritonavir for preventing and treating COVID-19. Cochrane Database Syst Rev 9:CD015395
351. Hashemian SMR, Sheida A, Taghizadieh M, Memar MY, Hamblin MR, Bannazadeh Baghi H, Sadri Nahand J, Asemi Z, Mirzaei H (2023) Paxlovid (Nirmatrelvir/Ritonavir): A new approach to Covid-19 therapy? Biomed Pharmacother 162:114367
352. Tian F, Chen Z, Feng Q (2023) Nirmatrelvir-ritonavir compared with other antiviral drugs for the treatment of COVID-19 patients: A systematic review and meta-analysis. J Med Virol 95:e28732
353. Tian H, Yang C, Song T, Zhou K, Wen L, Tian Y, Tang L, Xu W, Zhang X (2023) Efficacy and safety of paxlovid (nirmatrelvir/ritonavir) in the treatment of COVID-19: An updated meta-analysis and trial sequential analysis. Rev Med Virol e2473
354. Amani B, Amani B (2023) Efficacy and safety of nirmatrelvir/ritonavir (Paxlovid) for COVID-19: A rapid review and meta-analysis. J Med Virol 95:e28441
355. Amani B, Akbarzadeh A, Amani B, Shabestan R, Khorramnia S, Navidi Z, Rajabkhah K, Kardanmoghadam V (2023) Comparative efficacy and safety of nirmatrelvir/ritonavir and molnupiravir for COVID-19: A systematic review and meta-analysis. J Med Virol 95:e28889
356. Lei S, Chen X, Wu J, Duan X, Men K (2022) Small molecules in the treatment of COVID-19. Signal Transduct Target Ther 7:387
357. McCarthy MW (2022) Optimizing the use of Paxlovid in clinical practice. Drugs Today 58:539–546
358. Navitha Reddy G, Jogvanshi A, Naikwadi S, Sonti R (2023) Nirmatrelvir and ritonavir combination: an antiviral therapy for COVID-19. Expert Rev Anti Infect Ther 1–13
359. Zheng Q, Ma P, Wang M, Cheng Y, Zhou M, Ye L, Feng Z, Zhang C (2023) Efficacy and safety of Paxlovid for COVID-19:a meta-analysis. J Infect 86:66–117
360. Rosenberg K (2022) New Data on Paxlovid Reported. Am J Nurs 122:59
361. Weng C, Xie R, Han G, Yuan Y, Li S, Wang C, Wang X, Jiang W, Jiang L (2023) Safety and Efficacy of Paxlovid Against Omicron Variants of Coronavirus Disease 2019 in Elderly Patients. Infect Dis Ther 12:649–662
362. Niraj N, Mahajan SS, Prakash A, Sarma P, Medhi B (2022) Paxlovid: A promising drug for the challenging treatment of SARS-COV-2 in the pandemic era. Indian J Pharmacol 54:452–458.
